# Association between *CRP* rs1800947 genotypes, dexamethasone use, postoperative CRP level and morbidity in adult cardiac surgical patients in post-hoc analysis of the observational INFLACOR cohort trial

**DOI:** 10.1038/s41598-026-54801-9

**Published:** 2026-05-24

**Authors:** Maciej Michał Kowalik, Romuald Lango, Maciej Brzeziński, Magdalena Chmara, Andrzej Łoś, Magdalena Łasińska-Kowara, Piotr Siondalski, Dariusz Jagielak, Jan Rogowski

**Affiliations:** 1 Department of Anesthesiology and Intensive Care, Medical University of Gdańsk, ul. Dębinki 7, Gdańsk, 80-211 Poland; 2 Department of Cardiac Anesthesiology, Medical University of Gdańsk, Gdańsk, Poland; 3 Department of Cardiac and Vascular Surgery, Medical University of Gdańsk, Gdańsk, Poland; 4 Centre of Translational Medicine, Medical University of Gdańsk, Gdańsk, Poland

**Keywords:** Cardiac surgery, Adults, Cardiopulmonary bypass, C-reactive protein, Rs1800947, Dexamethasone, Inflammation, Morbidity, Genetic association study, Genetics, Biomarkers, Diagnostic markers, Predictive markers, Prognostic markers

## Abstract

Postoperative inflammation contributes to organ dysfunction and complications after cardiac surgery. C-reactive protein (CRP) is a common marker of systemic inflammation. In this study, we assessed how postoperative CRP levels and their trajectories are associated with the *CRP* rs1800947 gene variant and perioperative dexamethasone use, and how these factors relate to postoperative morbidity. We analysed CRP levels in 484 adults who underwent cardiac surgery with cardiopulmonary bypass (CPB). CRP levels on postoperative day 1 (CRP1) were compared across the rs1800947 genotype and intraoperative dexamethasone dose: (1) none, (2) low (median 0.4 mg/kg), or (3) high (median 1.0 mg/kg). The 4-day CRP trajectories were evaluated in a subgroup of 174 patients with serial CRP measurements. The composite outcome of 30-day morbidity and mortality was analysed to reveal the association with CRP trajectories. Results: Adjusted analysis revealed an association between the GC genotype and a 15% reduction in CRP1 levels. Dexamethasone, regardless of dose, reduced CRP1 levels by ~ 30%. Morning surgery increased CRP1 levels by 27%. Elevated preoperative CRP levels increased postoperative CRP1 levels by 19%. CRP trajectories varied by genotype and dexamethasone exposure. Patients were grouped into four trajectory types, whose associations with postoperative complications differed significantly. Conclusions: Postoperative CRP levels and trends were associated with genetic and clinical factors. Low-dose dexamethasone is as effective as high-dose dexamethasone in limiting increases in CRP1 level. The CRP patterns associated with CRP genotypes and dexamethasone use potentially represent inflammatory endotypes linked to different morbidity risks.

Trial registration: This study was registered in the US National Clinical Trial under the code NCT01020409 https://clinicaltrials.gov/ct2/show/study/NCT01020409. Registered on November 24, 2009.

**Supplementary Information:**

The online version contains supplementary material available at 10.1038/s41598-026-54801-9.

## Introduction

Most cardiac surgeries are performed using cardiopulmonary bypass (CPB), a significant contributor to systemic inflammatory response syndrome (SIRS) because of its direct effect on white blood cells and platelets^1^. This response can be triggered by not only the surgical procedure and CPB itself but also by factors such as reperfusion injury, transfusions, and anaphylactic reactions. The resulting inflammation involves multiple molecular and cellular pathways, closely resembling the host response observed in cases of infectious sepsis, and leads to widespread changes in gene expression, both activation and suppression^1,2^. This syndrome is characterized by clinical symptoms such as mild fever, tachycardia, vasoplegia, and hypotonia and laboratory findings such as leukocytosis and elevated acute phase proteins and inflammation mediators, with C-reactive protein (CRP) being the most common marker for distinguishing between infectious and non-infectious causes of SIRS^3–6^.

SIRS is recognized as a major pathophysiological mechanism underlying early postoperative complications following cardiac surgery, including coagulation disorders, postoperative delirium, vasoplegia, acute kidney injury, acute lung injury, and atrial fibrillation^1^. Although advances in the materials and design of CPB circuits have reduced their potential to activate inflammation, CPB remains a key contributor to early postoperative morbidity^7^. The significance of this inflammatory response has been recognized since the early days of cardiac surgery^8^. While inflammation is integral to wound healing, excessive systemic inflammatory response syndrome is unfavourable, potentially triggering secondary organ dysfunction following CPB^1,9^. Efforts to reduce the duration of CPB or avoid it altogether have led to the development of off-pump coronary artery bypass grafting (OP-CABG), which has resulted in fewer postoperative complications in elderly patients, although its long-term benefits remain debated^10,11^. Since the early days of cardiac surgery, systemic inflammation has been a target of therapeutic intervention. Once part of standard cardiac anaesthesia care, perioperative corticosteroid use represents another strategy to mitigate systemic inflammation^12–14^. Corticosteroids are potent anti-inflammatory agents that have long been used in this context^1,12,14–17^. These drugs have complex effects, including the modulation of gene expression and translation^18^. Dexamethasone and prednisolone are the most commonly used corticosteroids for cardiac surgery. However, large multicentre randomized trials have sparked debate regarding their use, particularly at high doses. Although some benefits have been observed, such as reduced postoperative complications in patients with chronic obstructive pulmonary disease, high-dose corticosteroids have also been linked to adverse effects, including pleural effusions^18,19^. These findings appear to contrast with early studies supporting the use of corticosteroids to attenuate postoperative inflammation^12–14^. Given the improved biocompatibility of CPB circuits, the role of corticosteroids was reassessed in two major trials: DECS and SIRS^1,7,18,19^. Despite their large-sized samples, neither trial demonstrated a reduction in early morbidity nor mortality^18,19^. Both trials evaluated relatively high corticosteroid doses equivalent to approximately 2000–2500 mg of hydrocortisone. Differences in pharmacokinetics are also relevant: dexamethasone has a half-life exceeding 30 h, whereas the half-life of prednisolone ranges from 18 to 36 h^20^. At low doses, dexamethasone is a well-established adjuvant to general anaesthesia, effectively reducing the likelihood of postoperative nausea and the analgesic demand^21,22^. However, the optimal dosing, duration, and personalized indications for corticosteroid use in cardiac surgery settings remain unclear. Owing to ongoing controversy, recent guidelines, such as the 2019 Enhanced Recovery After Cardiac Surgery (ERACS) recommendations and the updated 2022 French guidelines, lack clear recommendations for corticosteroid use in adult patients^23,24^. Recent meta-analyses of paediatric cardiac surgical studies suggest that corticosteroid use reduces the duration of mechanical ventilation and probably decreases the incidence of low cardiac output syndrome and reoperations; however, it does not significantly reduce all-cause mortality^25,26^. Notably, the inconclusive results of the DECS and SIRS trials may reflect unaccounted genetic and biological heterogeneity among patients.

Genetic variation is increasingly recognized as an important factor influencing postoperative outcomes and patterns of inflammation. Genome-wide association studies (GWAS), such as those conducted within the Perioperative Genetics and Safety Outcome Study (PEGASUS), revealed several single-nucleotide polymorphisms (SNPs) associated with postoperative morbidities in patients undergoing on-pump cardiac surgery and provided broad genetic insight into the regulation of blood CRP levels^27–32^. Given the complex nature of these complications, polygenic risk models have been proposed to enhance the prediction accuracy for adverse outcomes^33,34^. Among the 266 recently identified genetic loci is also the *CRP *rs1800947 genotyped in the PEGASUS trial^28,32^. This synonymous mutation on chromosome 1q23.2 has been associated with lower serum CRP levels in patients undergoing coronary artery bypass grafting (CABG) and in European patients with myocardial infarction^35,36^. The GC genotype was also associated with lower CRP levels in patients with autoimmune diseases such as lupus and Crohn’s disease^37,38^.

**Fig. 1:**
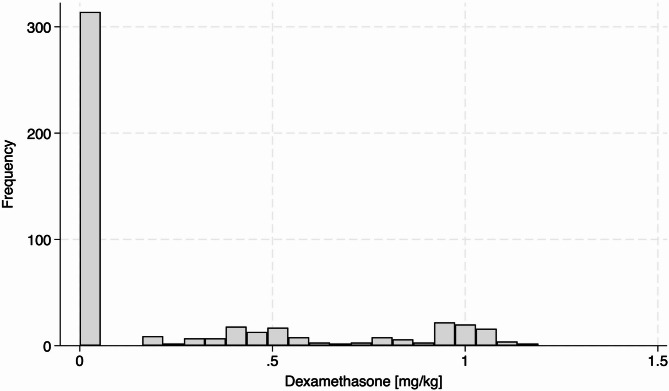
Distribution of dexamethasone doses per kilogram of body weight in 484 patients from the analysed INFLACOR cohort.

The prospective observational INFLACOR trial, aimed at assessing associations between selected SNPs, cytokines and postoperative morbidities triggered by systemic inflammation, was inspired by early research by a group at Durham University^39^. The available methods in 2008, when INFLACOR was designed and planned, allowed for a candidate gene association study, and the haplotypes associated with CRP regulation were identified thereafter, thus the only SNP associated with CRP regulation genotyped in the INFLACOR study was rs1800947. Because there were no clear guidelines on dexamethasone use in 2008, during the INFLACOR trial, dexamethasone dosing was performed at the discretion of the attending anaesthesiologist. The doses were typically rounded to full ampules (4 mg or 8 mg), resulting in some inter-patient variation, but three distinct dosing clusters could be clearly identified. (Fig. 1)

This provided a unique variance of dexamethasone doses, clearly concentrating in three clusters: no dexamethasone, approximately 0.4 mg/kg, and 1.0 mg/kg.

In this context, we hypothesized that postoperative CRP levels are influenced not only by known surgical factors but also by the *CRP* rs1800947 genotype and dexamethasone administration. To evaluate these hypotheses, we performed a retrospective analysis of the INFLACOR dataset with the aim of evaluating the associations between *CRP* genotypes, different dexamethasone doses, postoperative CRP levels, and host responses defined by postoperative morbidity.

## Methods

### Study design

This study presents a post hoc analysis of serum C-reactive protein (CRP) levels following cardiac surgery with cardiopulmonary bypass (CPB) using data from an observational INFLACOR (INFLAmmation After Cardiac OpeRations) cohort study conducted at a tertiary university hospital. This translational study included 525 adult male and female patients. Clinical data were collected during active recruitment between October 2009 and April 2011^39^. The inclusion criteria were as follows: (1) age ≥ 18 years, (2) first-time elective cardiac surgery with CPB, and (3) written informed consent. The exclusion criteria were as follows: (1) history of previous open-chest cardiac surgery, (2) emergency or urgent procedures, (3) off-pump cardiac surgery, and (4) refusal to participate. The INFLACOR study protocol, which included the enrolment of human subjects, the collection of biological material, the collection of clinical data and the performance of all laboratory tests, was designed in accordance with the Helsinki Declaration and relevant guidelines and regulations and was approved by the Independent Bioethics Commission of Research at the Medical University of Gdańsk (NKEBN/358/2007) and registered in the National Clinical Trial database (NCT01020409, registered 24/11/2009; https://clinicaltrials.gov/ct2/show/study/NCT01020409). Every patient in the study signed an informed consent form to participate in the trial.

### Outcome measures

The primary outcome was serum CRP concentration measured 12–18 h after the completion of surgery (postoperative day 1, CRP1). Patients with missing CRP1 measurements were excluded. Longitudinal CRP trajectories were analysed in a subgroup analysis that included only patients for whom at least three out of four CRP values were available during the first four postoperative days. To assess the effects of genotype and dexamethasone dosing, CRP1 levels were compared between the different rs1800947 genotypes across three dexamethasone strata, with a single dose administered at the induction of anaesthesia: group 1 - no dexamethasone; group 2 - low dose (median 0.4 mg/kg; range 0.2–0.69); and group 3 - high dose (median 1.0 mg/kg; range 0.7–1.2).

### Secondary outcomes

Secondary outcome measures included composite morbidity (death, myocardial infarction, stroke, renal failure, or respiratory failure within 30 days, as defined by Dieleman et al^18^.), hours on ventilation (HOV), length of ICU stay (ICU-LOS), total length of hospital stay, and 30-day and 5-year mortality rates.

### Genotyping and confounder analysis

As a candidate gene association study with a targeted inclusion of 500 patients, the INFLACOR study accommodated the following SNP selection criteria for genotyping and analysis: (1) a previously reported minor allele frequency of ≥20% and (2) a reported biological effect of > 5%. Information on specimen collection, handling, storage, extraction, laboratory methods, genotyping of *CRP *rs1800947 and genetic data quality control was published elsewhere^39^. Participants and investigators performing genetic, cytokine, and laboratory testing were blinded to clinical data and outcome measures. The selection of potential confounders was guided by clinical experience and the literature^36^. Candidate variables included age, sex, operation time of day, body mass index (BMI), presence of infective endocarditis, chronic corticosteroid use, statin therapy, elevated preoperative CRP (> 5 mg/L), impaired kidney function (eGFR < 60 mL/min/m²), CPB duration, intraoperative hypotension, serum lactate levels, and transfusion of red blood cell concentrates (RBCCs).

Preliminary findings from this dataset were presented at the 34th Annual Meeting of the European Association of Cardiothoracic Anesthesiologists (EACTA) in Ghent, Belgium (September 4–6, 2019), S16:03, *“Comparison of effects of no-*,* medium-*,* and high-dose dexamethasone in adult cardiac surgery - A post hoc analysis of the prospective*,* observational INFLACOR trial”*, published in *Journal of Cardiothoracic and Vascular Anesthesia*, 33 S2 (2019) S92–S93.

### Statistical analysis

The sample size of the INFLACOR trial (*n* = 525), a prospective observational cohort study, was determined on the basis of the following: (1) the duration of grant funding (3 years), (2) financial limits that restricted laboratory analyses to no more than 500 patients, (3) the operating volume of the research centre, and (4) an assumed drop-out rate of 5%.

Continuous variables, including serum CRP1 levels, were tested for normality using the Shapiro-Wilk test. Depending on the data distribution and number of comparison groups, CRP1 levels were analysed using Student’s t-test, the Mann-Whitney test, the Kruskal-Wallis (KW) test, or the Wald chi-square test with James’ approximation. Categorical variables were compared using the Mantel-Haenszel test, Fisher’s exact test or Pearson’s chi-square tests. Linear regression was used to analyse the associations between CRP1 levels and confounding variables. Confounders that were significantly associated with CRP1 levels were included in a multiple linear regression model. Longitudinal CRP trends over the first four postoperative days were evaluated using a random-effects linear regression model with maximum likelihood estimation (MLE). Trends were analysed using the Cochran-Armitage (CA) exact test. All tests were two-sided, with a p-value of ≤ 0.05 required to reject the null hypothesis. Analyses were performed using licenced STATA/BE software (StataCorp LLC, College Station, TX, USA).

The different size of the dexamethasone groups introduced a potential selection bias; however, this was addressed via analysis of demographic and procedural data across the groups which did not show any differences. (Supplementary Material, Table S1). Another source of selection bias was the exclusion criteria of the study. Attrition bias is highly possible in the comparison of combined morbidity between the “longitudinal subgroup” (LS) and the rest of the cohort, as the LS was treated longer in the ICU, where surveillance and recording of outcomes were more effective. Efforts to reduce this bias were implemented during the active recruitment phase of the study and included training of the medical personnel involved in patient care and data recording, but some outcomes may not have been reported.

## Results

### Baseline characteristics

**Fig. 2:**
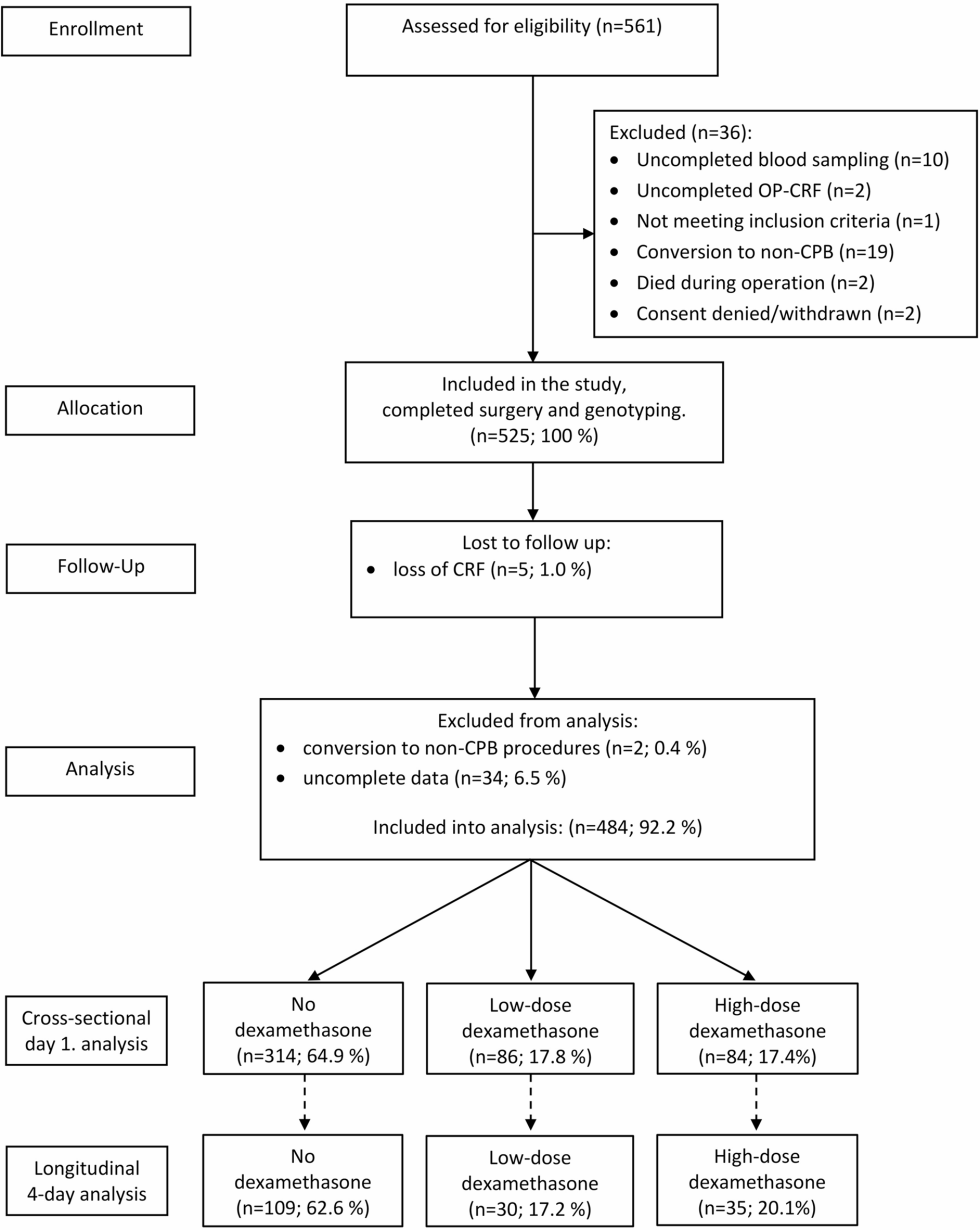
Patient flow in the INFLACOR cohort and reasons for exclusion.

Among the 561 patients invited to participate in the study, 525 (100%) underwent surgery and were genotyped. Among these, 484 (92.2%) met the inclusion criteria and were included in the current analysis. The patient flow and reasons for exclusion are illustrated in Fig. 2.

The INFLACOR cohort included patients who underwent a broad range of cardiac surgical procedures. The case mix and baseline demographics are presented in Table 1. The surgical procedures were grouped into six categories on the basis of complexity and increasing average CPB duration. As elective coronary artery bypass grafting (CABG) is routinely performed off-pump at the study centre, acute cases were excluded, and isolated CABG procedures were not represented in the cohort. However, CABG performed as part of complex or aortic procedures was included. Operations categorized as “other” had the shortest CPB times, whereas those involving multiple valves and/or additional procedures were the longest.

The minor allele (C) of rs1800947 had a frequency of 9.0%. Patients with the GC genotype had only a significantly lower BMI than those with the GG genotype did (median 25 vs. 27; *p* = 0.007; Supplementary Materials, Table S2). Dexamethasone use was balanced across genotypes, with no significant differences. The baseline characteristics were also well balanced across the three dexamethasone dose groups (Supplementary Materials, Table S2).

**Table 1:** Baseline characteristics of the 484 patients from the INFLACOR cohort.

Characteristics	
Demographic	
Age [years] mean ±SD	65 ±11
Sex [n] (%) Female Male	216 (45) 268 (55)
BMI [kg/m^2^] mean ±SD	27.5 ±5.0
Surgical procedure [n] (%)	
Other	15 (3)
Single valve	190 (39)
Aorta surgery	41 (8)
Single valve + other	152 (31)
Two valves	61 (13)
2–3 valves and/or other	25 (5)
***CRP*** rs1800947 genotypes [n] (%)	
GG GC	397 (82) 87 (18)
Dexamethasone use [n] (%)	
None	259 (65)
Low dose	67 (17)
High dose	71 (18)

## Primary outcome

### Day 1. cross-sectional analysis

The mean CRP1 concentration was 55.6, the median was 51.5, the range was 6–187, and the variance was 818.5 mg/dL. The wild GG genotype of the rs1800947 SNP was genotyped in 397 patients (82%), and the recessive GC genotype was genotyped in 87 patients (18%). The minor allele (C) frequency was 9%. CRP1 levels differed significantly according to genotype. In the unadjusted analysis, compared with GG carriers, GC carriers had 25–27% lower CRP1 levels across all dexamethasone groups. (Table 2).

**Table 2:** CRP1 levels (mg/dL), medians and interquartile ranges (IQRs), according to the *CRP* rs1800947 genotype and dexamethasone dose.

rs1800947 genotype Dexamethasone dose	CRP1 in GG (*n* = 397)	CRP1 in GC (*n* = 87)	*p*-value
None	64 (49–79)	48 (37–65)	< 0.0001
Low-dose (0.4 mg/kg)	37 (24–53)	27 (19–38)	0.14
High-dose (1.0 mg/kg)	37 (28–48)	28 (23–35)	0.04

Footnote: the *p* value is for the Mann-Whitney test.

**Fig. 3:**
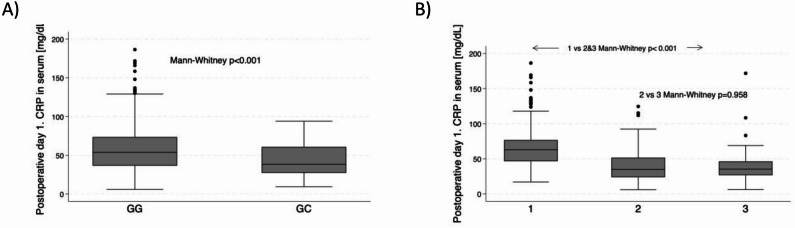
CRP levels on postoperative day 1 by genotype (Panel A) and dexamethasone exposure (Panel B).

Significant differences in CRP1 levels were observed in the genotype groups after treatment with either no-dexamethasone or high-dose dexamethasone but not low-dose dexamethasone in the unadjusted analysis. CRP1 levels in patients receiving dexamethasone, regardless of dose, were approximately 44% lower than those in patients who did not receive the drug. (Fig. 3)

The preoperative level of CRP (CRP0) was measured only in 91/484 (18.8%) patients. Preoperative CRP (CRP0) and delta CRP1-CRP0 did not differ significantly among genotypes in either the unstratified comparison of dexamethasone use or the stratified comparison of delta CRP, probably because the number of patients in the subgroups was too small.

Footnote: Panel A – GG/GC are genotypes of rs1800947 SNP; the *p* value is for the Wilcoxon rank-sum test. Panel B – 1 – patients not receiving dexamethasone, 2 – patients receiving 0.4 mg/kg dexamethasone, and 3 – patients receiving 1.0 mg/kg dexamethasone.

### Analysis of confounders

The univariate linear regression revealed that five of the 15 candidate variables were associated with CRP1 level: *CRP* rs1800947 genotype, BMI, dexamethasone use, morning surgery, and elevated preoperative CRP level. (Supplementary Materials, Table S3) Neither preoperative medication and comorbidities, nor CPB duration and blood product transfusion was associated with CRP1 levels. However, for hypotonia and lactate, both of which are factors considered indicators of intraoperative organ hypoperfusion, the *p*-values were 0.07 and 0.06, respectively. In the multiple linear regression, four of the five associated variables remained significant in the final model. The variance was 40.4% for the model including the *CRP* genotype and 37.2% for the model without the genotype, and the delta was 3.2%. (Table 3).

**Table 3:** Multiple linear regression model of variables associated with CRP level on postoperative day 1 (*n* = 484).

Variable	β_1_-coefficient	95% CI	Std. err.	*p*
*CRP* rs1800947 (GC vs. GG)	−13.6	−18.7 −8.4	2.6	0.000
Dexamethasone (Yes)	−27.1	−31.2 −22.9	2.1	0.000
Morning operation (Yes)	24.9	29.0 -20.8	2.1	0.000
Preoperative CRP > 5 mg/dL (Yes)	17.0	25.6 -8.5	4.4	0.000

Footnote: Intercept constant = 91.4 mg/dL; Std. err. – standard error; CI – confidence interval of β_1_; dexamethasone (Yes) – any dose of dexamethasone administered before the start of the operation.

### Adjusted effects (relative change)

On the basis of the multiple regression model, the adjusted effects on CRP1 levels [mg/dL] of the four identified predictor variables were as follows: (1) GC genotype: − 15% CRP1; (2) dexamethasone: − 30% CRP1; (3) morning surgery: + 27% CRP1; and (4) elevated preoperative CRP > 5 mg/dL: + 19% CRP1.

### Longitudinal analysis

**Fig. 4:**
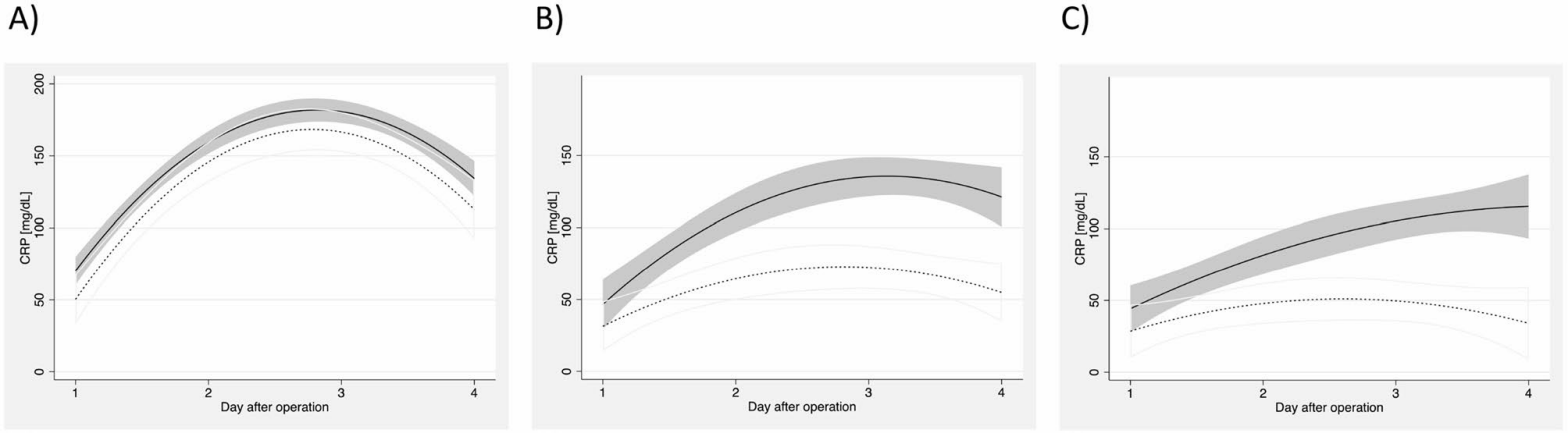
CRP trajectories (mean and 95% CI) during the first four postoperative days, stratified by the *CRP* rs1800947 genotype and dexamethasone dose. Solid line – patients with the *CRP* rs1800947 (GG) genotype; scattered line – patients with the *CRP* rs1800947 (GC) genotype; Panel A: patients not receiving dexamethasone; Panel B: patients receiving 0.4 mg/kg dexamethasone; Panel C: patients receiving 1.0 mg/kg dexamethasone.

CRP trajectory data for the first four postoperative days were available for 174 patients (36%). Six patient groups were defined based on dexamethasone doses and rs1800947 genotypes: (1) GG/no dexamethasone (*n* = 92), (2) GC/no dexamethasone (*n* = 17), (3) GG/0,4 mg/kg dexamethasone (*n* = 23), (4) GC/0,4 mg/kg dexamethasone (*n* = 7), (5) GG/1,0 mg/kg dexamethasone (*n* = 1), and (6) GC/1,0 mg/kg dexamethasone (*n* = 4). This analysis has two important drawbacks: (1) because of early ICU discharge practices, this subgroup included patients who were undergoing more complex or complicated procedures, and (2) while most of the day groups had complete data on day 1, a considerable amount of data were missing by day 4, especially for the GG/ High-dose (37% missing), GC/No-dexamethasone (29.4%), and GC/High-dose (50%) groups. (Supplementary Materials, Figure S1) The patients in the longitudinal analysis subgroup were significantly older, had higher EUROSCORE II scores, underwent more complex surgical procedures, had longer CPB times, higher day 1 APACHE III scores, and significantly more HOV, longer ICU-LOS, longer HOS-LOS, and higher 30-day mortality rates. (Supplementary Material, Table S4). In a random-effects linear regression model, CRP levels were consistently lower in GC carriers regardless of dexamethasone use (*p* < 0.001; Supplementary Material, Table S5). However, quadratic function trajectory patterns varied by dexamethasone dose: (A) No dexamethasone: “broken-stick” pattern, peaking on days 2–3 in both genotypes). (B) Low dose: similar shape but lower levels, particularly in those with the GC genotype. (C) High dose: continuous increase in those with the GG genotype; low-dose-like pattern in those with the GC genotype. (Fig. 4)

### Secondary outcomes

#### Morbidity and mortality analysis

The median LOS in the ICU was 1.7 days (IQR: 0.9–2.9; range 0.2–29.7), the median LOS in the hospital was 8 days (IQR: 7–11; range: 5–54), the 30-day mortality was 17 (3.2%), and the 5-year mortality was 113 (23.4%). Composite morbidity (death, MI, stroke, and renal or respiratory failure within 30 days) occurred in 68/484 patients (14.1%). CRP1 levels did not differ significantly: 49 mg/dL (IQR: 34–45) in complicated cases vs. 52 mg/dL (IQR: 35–71) in uncomplicated cases (*p* = 0.26). The genotype distributions were similar: uncomplicated GC/GG = 74 (85.1%)/342 (86.2%) and complicated GC/GG = 13 (14.9%)/55 (13.9%) (*p* = 0.79). Additionally, none of the secondary outcomes (HOV, ICU/hospital LOS or mortality) were significantly associated with CRP1 level, genotype, or dexamethasone dose. Composite morbidity was strongly associated with 5-year survival (*p* < 0.001; Supplementary Material, Figure S2). Dexamethasone use was linked to a nonsignificant trend towards more complications in the entire cohort (*p* = 0.08; Supplementary Material, Figure S3).

#### Trajectory subgroup analysis

In the subgroup of 174 patients with trajectory data, 47 (20.8%) experienced complications versus 21/310 (11.7%) in the rest of the cohort (*p* < 0.001). Patients with CRP trajectories were grouped by similarity using correlation analysis (Supplementary Material; Figure S4) and visual assessment into four clusters:

- T1: no dexamethasone, GG or GC (*n* = 109).
- T2: GG with 0.4 mg/kg (*n* = 31).
- T3: GG with 1.0 mg/kg (*n* = 23).
- T4: GC with 0.4 or 1.0 mg/kg (*n* = 11).

**Fig. 5:**
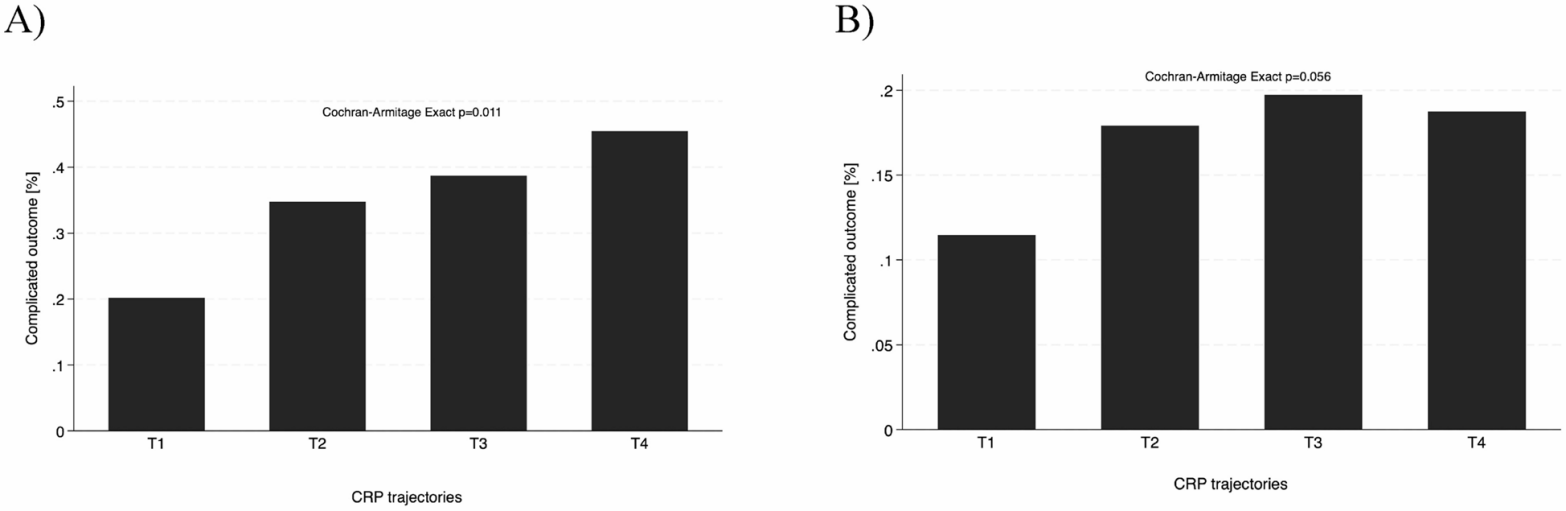
Frequency of composite morbidity (death, myocardial infarction, stroke, renal failure, or respiratory failure within 30 days) in four CRP trajectory clusters (T1–T4) defined by genotype and dexamethasone dose. Panel A shows 174 patients (the source group for CRP trajectory comparison), and Panel B shows the whole cohort (*n* = 484). T1 - patients with the GG or GC genotype who did not receive dexamethasone; T2 - patients with the GG genotype who received 0.4 mg/kg dexamethasone; T3 - patients with the GG genotype who received 1.0 mg/kg dexamethasone; T4 - patients with the GC genotype who received 0.4 mg/kg or 1.0 mg/kg dexamethasone.

The composite morbidity rates differed significantly between the four groups: T1, 20%; T2, 35%; T3, 39%; and T4, 45% (*p* = 0.01; Fig. 5).

A similar trend was observed in the full cohort, with a slightly lower complication rate in the T4 group. None of the other secondary outcomes differed significantly by trajectory in either cohort.

## Discussion

**Fig. 6:**
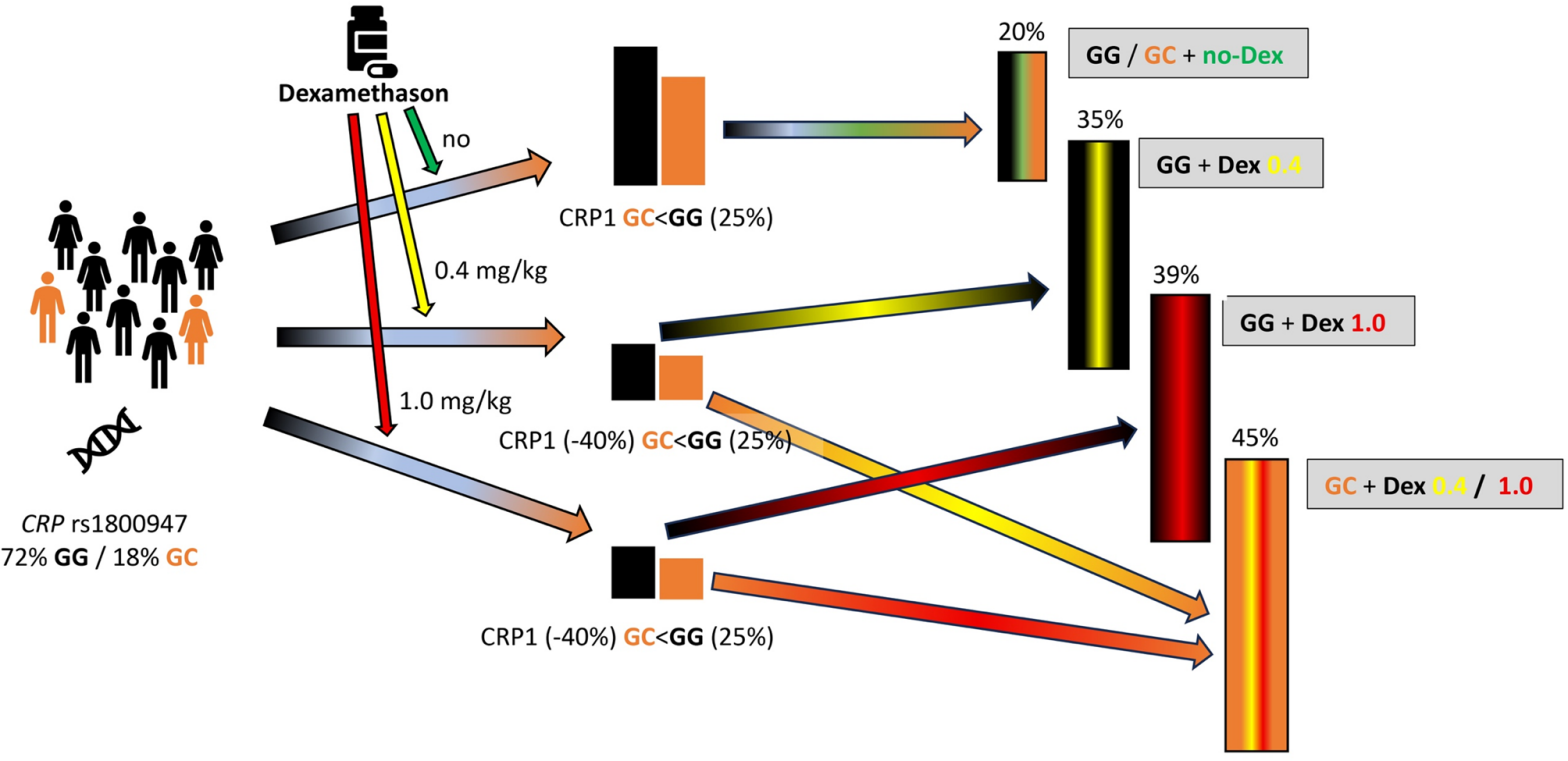
Univariate analysis revealing associations among *CRP* rs1800947 genotypes, dexamethasone use, postoperative day 1 CRP (CRP1) and composite outcome in the INFLACOR cohort of 484 patients who underwent cardiac surgery on cardiopulmonary bypass.

Legend: CRP – C-reactive protein; GG – wild genotype; GC – recessive genotype; GC < GG difference in CRP unadjusted for confounders; “no-Dex” – patients who did not receive dexamethasone (*N* = 314); “Dex 0.4” – patients who received 0.4 mg/kg dexamethasone (*N* = 86); “Dex 1.0” – patients who received 1.0 mg/kg dexamethasone; “Composite outcome” includes death, myocardial infarction, stroke, renal failure, or respiratory failure within 30 days after surgery.

The main findings of this study revealed that CRP levels on the first postoperative day (CRP1) after cardiac surgery on CPB were associated with both the *CRP* rs1800947 genotype and dexamethasone use. A novel finding in this study was that low-dose dexamethasone (0.4 mg/kg) reduced CRP1 levels as effectively as high-dose dexamethasone (1.0 mg/kg), with reductions of approximately 40% compared with levels observed in patients not receiving the drug—an effect consistent across both genotypes. (Fig. 6) A major drawback of the INFLACOR protocol was the lack of CRP measurement before the operation (CRP0). CRP0 data were available for only 19% of the cohort. On the basis of the primary findings, it seems logical that in a comparison of delta CRP1-CRP0, the effects of genotype and dexamethasone use should be accounted for. Unfortunately, neither the CRP0 nor the delta CRP1-CRP0 differed significantly between genotypes, most likely because the number of patients in the subgroups was too small. Therefore, the other significant results should be interpreted carefully and verified in a larger sample.

The minor allele frequency of the analysed *CRP*rs1800947 in our northern Polish cohort was 9.0% - similar to other cohorts from this part of Europe. Perry et al. previously reported an association between this SNP and postoperative CRP in CABG patients^36^. Our study extends these findings to a broader population of patients undergoing other types of cardiac surgeries on CPB.

An explorative univariate analysis confirmed that five variables were associated with CRP1: BMI, morning surgery, elevated preoperative CRP levels, dexamethasone use, and the *CRP*rs1800947 genotype. In this study, we confirmed previous findings that higher BMI and elevated preoperative CRP levels (> 5 mg/L) were associated with higher postoperative CRP levels^6,36,40^. In contrast, significant differences in CRP1 levels between morning and afternoon operations, which revealed how CRP levels increase after cardiac surgery on CPB, remained a significant covariate in the multiple logistic regression analysis. Surprisingly, none of the surgery related factors, such as CPB duration or RBCC transfusion, were associated with CRP1 levels.

While the limited size of the cohort implies that the results of the univariate analysis on the impact of confounding variables on CRP1 must be interpreted with caution, we performed a multiple linear regression. In the final model, four variables remained significantly associated and explained 40% of the CRP1 level variance: (1) the rs1800947 genotype, (2) use of any dose of dexamethasone, (3) preoperative CRP level and (4) morning surgery. BMI proved insignificant after adjusting for the other covariates. The *CRP* SNP accounted for approximately 3.2% of the variance. Given the complex, multifactorial regulation of inflammation, this contribution is modest but notable.

While large-scale GWAS have expanded our understanding of the polygenic regulation of CRP, our study focused on a single variant—*CRP*rs1800947—which proved an association between CRP blood levels in the acute, specific context of on-pump cardiac surgery and shed new light on postoperative inflammation^32^. No further haplotype analyses were performed in this study; notably, this SNP was reported in haplotypes associated with postoperative CRP levels in cardiac surgical patients in other studies^36^. These complementary approaches underscore the need to validate GWAS signals in physiologically relevant and outcome-linked models to inform patient-specific perioperative strategies.

Another novel aspect of our analysis is the characterization of four-day CRP trajectories. Although limited by selection bias towards more complex cases and small numbers of patients within the subgroups, the trajectory data revealed consistently lower CRP levels in GC genotype carriers and distinct trajectory patterns. Interestingly, in the high-dose dexamethasone group, the patients with the GG genotype exhibited a continuous increase in CRP level, whereas those who were GC carriers exhibited a flatter, attenuated course. These patterns suggest distinct inflammatory endotypes in the first postoperative days with different clinical implications because, importantly, the trajectory-based clusters were associated with postoperative composite morbidity. Notably, the composite morbidity rate in the INFLACOR cohort was nearly double that in the DECS study (14.1% vs. ~8%), likely because of the more diverse and higher-risk surgical population in INFLACOR, which included predominantly valve, aortic, and complex cases, whereas the DECS study included only CABG patients. Compared with CABG alone, valve surgery is associated with higher morbidity rates^41,42^. These data suggest that dexamethasone, particularly in patients with the GC genotype, undergoing complex procedures, may excessively blunt the inflammatory response, potentially contributing to compensatory anti-inflammatory response syndrome (CARS) and increased morbidity^43^. This hypothesis is supported by similar trends observed in the full cohort, lending biological plausibility. (Fig. 6) Unsurprisingly, 30-day composite morbidity was significantly associated with 5-year survival.

Our findings align with emerging approaches to sepsis, in which similar inflammatory endotypes influence outcomes and therapeutic responses^44,45^. While this analysis was limited to a single-centre non-randomized cohort and lacks external replication, the internal consistency of associations across genotypes, pharmacological modulation, CRP trajectories, and clinical outcomes support the biological plausibility of the findings. These data should be interpreted as hypothesis-generating and underscore the need for validation in independent cohorts. Further research is needed to determine whether these findings extend to other cardiac surgical populations and could be applied to patient-specific perioperative dosages of dexamethasone.

### Limitations

This study had several limitations. The INFLACOR cohort was relatively small for a genetic association study and lacked a comparative replication cohort. The study is imbalanced in terms of treatment intervention, resulting in small numbers of patients receiving corticosteroids and very small numbers of patients in the treatment groups with the CRP genotypes of interest. An important drawback is the lack of preoperative CRP data, preventing comparisons of CRP levels between genotypes before surgery and between delta values of CRP1-CRP0. Nevertheless, the consistency of the observed effects across time, strata (genotype, dexamethasone) and outcomes strengthens the internal validity of this study. The regression model revealed only a few covariates that were significantly associated with postoperative CRP1 levels, possibly because of the limited sample size. Only one SNP (*CRP*rs1800947) was analysed, although 265 other independent genetic loci were associated with CRP level and inflammation^32,36^. A substantial amount of important data were missing from the longitudinal analysis. Additionally, it was biased towards sicker patients who remained in the ICU longer. Nonetheless, the consistent findings in the broader cohort support the robustness of our observations.

## Conclusions

CRP levels after cardiac surgery with CPB are associated with the *CRP* rs1800947 genotype, dexamethasone use, surgical timing, BMI, and preoperative CRP level. The adjusted analysis revealed the following results:

- The GC genotype was associated with a 15% reduction in CRP1 level.
- Dexamethasone reduced CRP1 levels by 30%, regardless of dose.
- Morning surgery increased CRP1 levels by 27%.
- Elevated preoperative CRP levels increased CRP1 levels by 19%.

Low-dose dexamethasone (0.4 mg/kg) was as effective as high-dose dexamethasone (1.0 mg/kg) at reducing CRP1 levels. Four CRP trajectory patterns were identified by association with the genotype and dexamethasone dose. These endotypes are significantly associated with postoperative morbidity and may reflect biologically distinct inflammatory responses.

## Supplementary Information

Below is the link to the electronic supplementary material.

Supplementary Material 1

Supplementary Material 2

## Data Availability

All data contained in this article are available on request from the corresponding author.
