# Supplementary material for: Association between CRP rs1800947 genotypes, dexamethasone use, postoperative CRP level and morbidity in adult cardiac surgical patients in post-hoc analysis of the observational INFLACOR cohort trial
Source: Sci Rep. 2026 May 24;16:23754. doi: 10.1038/s41598-026-54801-9 (PMC13429688; doi:10.1038/s41598-026-54801-9)
Supplement: Supplementary file 1 — Supplementary Material 1 [file 41598_2026_54801_MOESM1_ESM.pdf]

Association between CRP rs1800947 genotypes, dexamethasone use, postoperative CRP level and morbidity in adult cardiac surgical patients in post-hoc analysis of the observational INFLACOR cohort trial.

Maciej Michał Kowalik et al.

Supplementary Materials

Table S1

Baseline characteristics of the 484 patients from the INFLACOR cohort between the three dexamethasone use strata: 1 – "no dexamethasone", 2 – "low-dose dexamethasone", and 3 – "high-dose dexamethasone".

Characteristics	Group 1 (n=314)	Group 2 (n=86)	Group 3 (n=84)	*p*-value
Demographic				
Age [years] median (IQR)	67 (58-74)	67 (61-71)	63 (59-74)	0.68[1]
Sex – male/female [n] (%)	176/138 (56/44)	53/33 (62/38)	39/45 (46/54)	0.13[2]
BMI [kg/m^2] median (IQR)	27 (24-31)	27 (24-30)	27 (22-30)	0.21[1]
Surgical procedure [n] (%)				
Other	11 (4)	3 (3)	1 (1)	0.13[2]
Single valve	126 (40)	33 (38)	31 (37)	
Aorta surgery	27 (9)	5 (6)	9 (11)	
Single valve + other	101 (32)	26 (30)	25 (30)	
Two valves	37 (12)	16 (19)	8 (10)	
2 or 3 valves and/or other	12 (4)	3 (3)	10 (12)	

Footnote: IQR, inter-quartile range; 1, Wald chi-square test with James' approximation; 2, Pearson's chi-square test.

Table S2

Comparison of baseline characteristics of the n=484 patients from the INFLACOR cohort according to the genotypes of *CRP* rs1800947 genotypes.

Genotypes<br>Characteristics	GG<br>(n=397; 82.0%)	GC<br>(n=87; 18.0%)	*p*-value
Demographic			
Age [years] median (IQR)	67<br>(59-74)	64<br>(57-72)	0.13[1]
Sex – male/female [n] (%)	221/176<br>(56/44)	47/40<br>(54/46)	0.78[2]
BMI [kg/m^2] median (IQR)	27<br>(24-31)	25<br>(23-30)	0.007[1]
Surgical procedure [n] (%)			
Other	12 (3)	3 (3)	0.93[2]
Single valve	153 (39)	37 (43)	
Aorta surgery	34 (9)	7 (8)	
Single valve + other	129 (32)	23 (26)	
Two valves	49 (12)	12 (14)	
2 or 3 valves and/or other	20 (5)	5 (6)	
Dexamethasone use [n] (%)			
No-dexamethasone	259 (65)	55 (63)	0.50[2]
Low-dose (0,4 mg/kg)	67 (17)	19 (22)	
High-dose (1,0 mg/kg)	71 (18)	13 (15)	

Footnote: IQR, inter-quartile range; 1, Mann-Whitney test; 2, Pearson's chi-square test.

Table S3

Results of linear regression analysis of potential confounding factors on postoperative day 1. CRP level.

Variable	Coefficient	95% CI	SE	t	p
Age	-0.15	-0,38 0.07	0.11	-1.34	0.180
Sex (Male – 1, Female -2)	-3.38	-8.52 1.76	2.61	-1.29	0.197
Morning hour operation beginning	-24.3	-29.2 -19.4	2.48	-9.77	**0.001**
BMI	0.88	0.37 1.38	0.26	3.40	**0.001**
Infective endocarditis	-0.25	-16.70 16.19	8.37	-0.03	0.976
Chronic corticosteroids	-4.27	-21.43 12.89	8.73	-0.49	0.625
Statin therapy	-1.45	-6.56 3.66	2.60	-0.56	0.578
Preoperative CRP >5 mg/dL	-8.94	-18.19 0.32	4.71	-1.90	**0.050**
CKD	3.61	-2.04 9.26	2.88	1.26	0.209
CPB duration [Min]	0.02	-0.03 0.07	0.03	0.61	0.569
RBCC transfusion	-0.88	-2.20 0.44	0.67	-1.31	0.192
Hypotonia [Min]	-0.15	-0.31 0.01	0.08	-1.81	0.071
Lactates [mmol/dL]	-2.98	-6.10 0.14	1.59	-1.88	0.061
CRP rs1800947 [GC>GG]	-14.23	-20.76 -7.69	3.33	-4.28	**0.000**
Dexamethasone [No>Yes]	-25.51	-30.35 -20.66	2.47	-10.34	**0.000**

Abbreviations: BMI, body mass index; CKD, chronic kidney disease (eGFR <60 ml/min/m20; RBCC, red blood cells concentrate; hypotonia, MAP <60 mmHg

Table S4

Comparison of patients included in the longitudinal analysis (n=174) with the rest of the INFLACOR cohort (n=310).

Characteristics	Longitudinal analysis (n=174)	Other patients (n=310)	*p*-value
Age [years] median (IQR)	67 (60 – 74)	65 (57 – 73)	0.024[1]
EUROSCORE II median (IQR)	4.92 (3.32 – 8.9)	4.29 (2.27 – 6.76)	<0.001[1]
CPB time [Minutes] median (IQR)	142 (115 – 184)	117 (95 – 150)	<0.001[1]
Surgical procedure [n] (%)			
Other	3 (20)	12 (80)	<0.001[2]
Single valve	48 (25)	142 (75)	
Aorta surgery	15 (37)	26 (63)	
Single valve + other	60 (40)	92 (60)	
Two valves	31 (51)	30 (49)	
2 or 3 valves and/or other	17 (68)	8 (32)	
APACHE III – day 1 [score] median (IQR)	37 (30 – 45)	31 (22 – 38)	<0.001[1]
HOV [hours] median (IQR)	17 (11 – 24)	10 (8 – 15)	<0.001[1]
ICU-LOS [days] median (IQR)	3 (2 – 5)	1 (1 – 2)	<0.001[1]
HOS-LOS [days] median (IQR)	9 (7 – 12)	8 (7 – 10)	<0.001[1]
30-day mortality [n] (%)	12 (6.9)	5 (1.6)	0.002[2]

CPB, cardiopulmonary bypass; HOV, hours on ventilator; ICU-LOS, ICU length of stay; HOS-LOS, hospital length of stay; 1, Mann-Whitney test; 2, Pearson's chi square test

Table S5

The random-effects ML linear regression model for longitudinal analysis.

Number of obs = 606; Number of groups = 174; Random effects u_i ~ Gaussian

Obs per group: min = 2; avg = 3.5; max = 4; LR chi2(2) = 71.29

Log likelihood = -3335.1692; Prob > chi2 = 0.0000

	Coefficient	Std. err.	z	P>|z|	95% CI
CRPgenecode	-28.46855	7.487473	-3.80	0.000	-43.14373 -13.79337
Dexametkat	-29.39488	3.432781	-8.56	0.000	-36.12301 -22.66675
_cons	193.2838	10.68458	18.09	0.000	172.3424 214.2252
/sigma_u	19.77665	3.717018			13.68263 28.58485
sigma_e	56.47039	1.895778			52.87434 60.31101
rho	.1092496	.0395326			.0500239 .2071255

LR test of sigma_u=0: chibar2(01) = 9.24 Prob >= chibar2 = 0.001

Figure S1

Missing data in longitudinal analysis

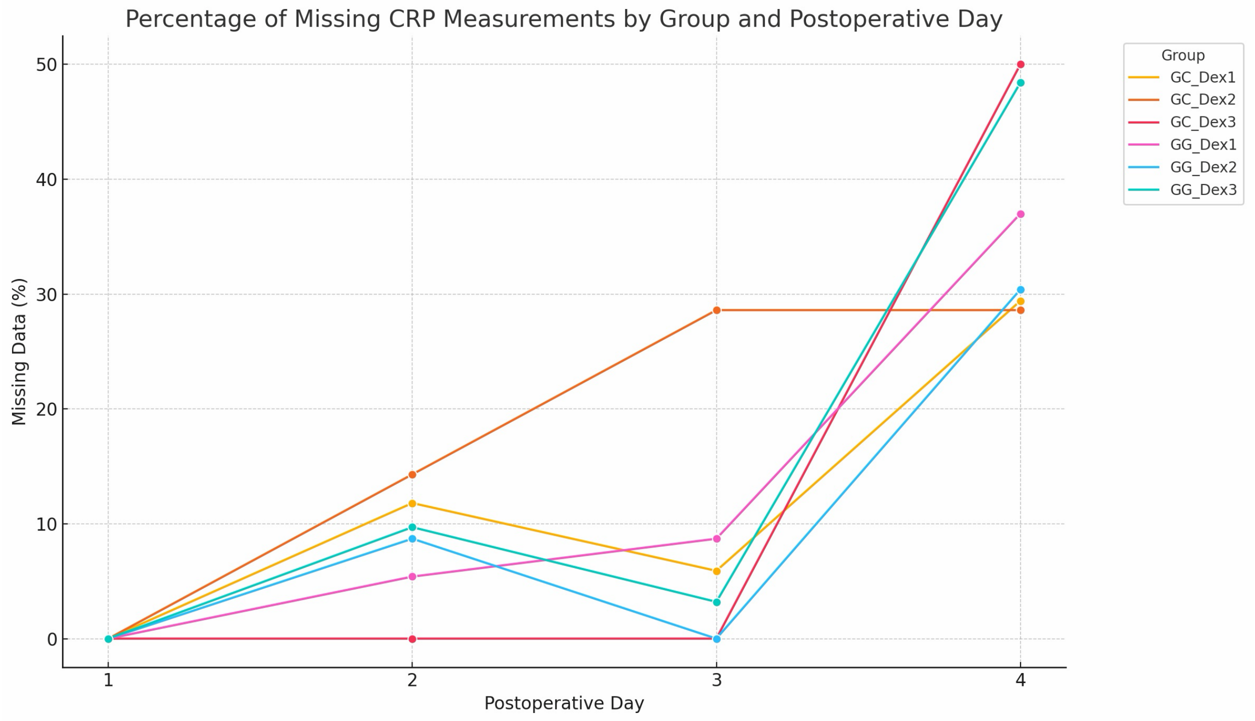

Figure S2

Kaplan-Meier 5-year survival curves for 484 patients with complicated and non-complicated postoperative course.

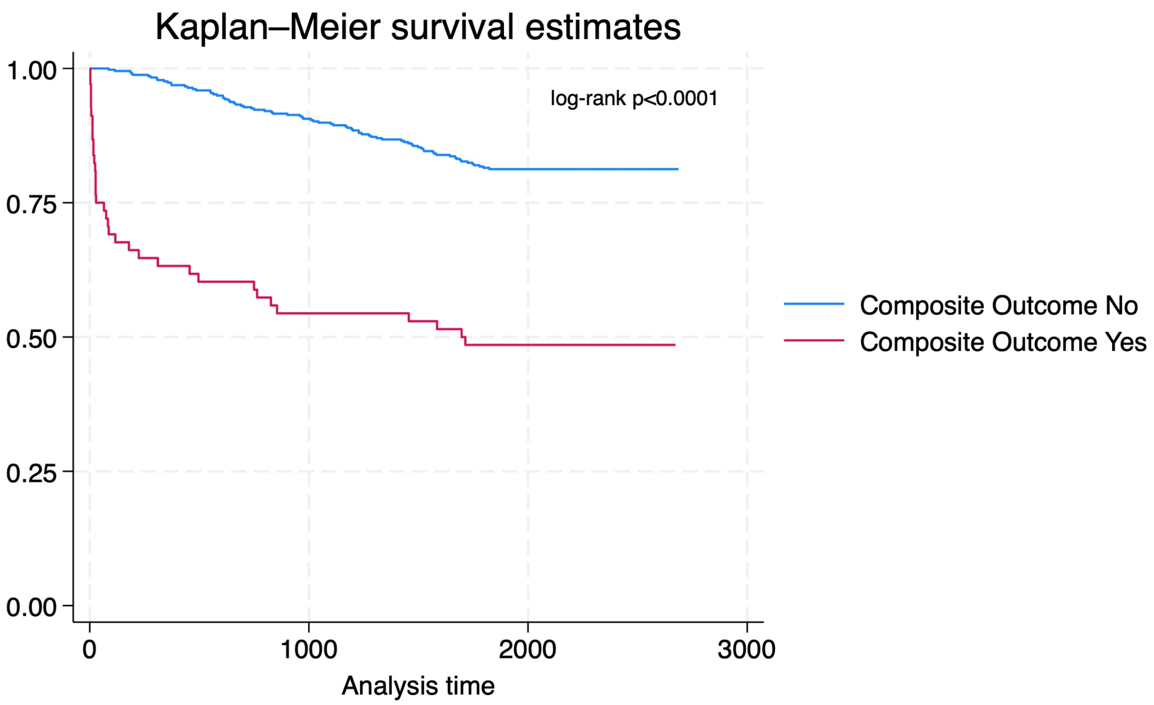

Figure S3

Frequencies of the composite morbidity and dose of dexamethasone in 484 patients.

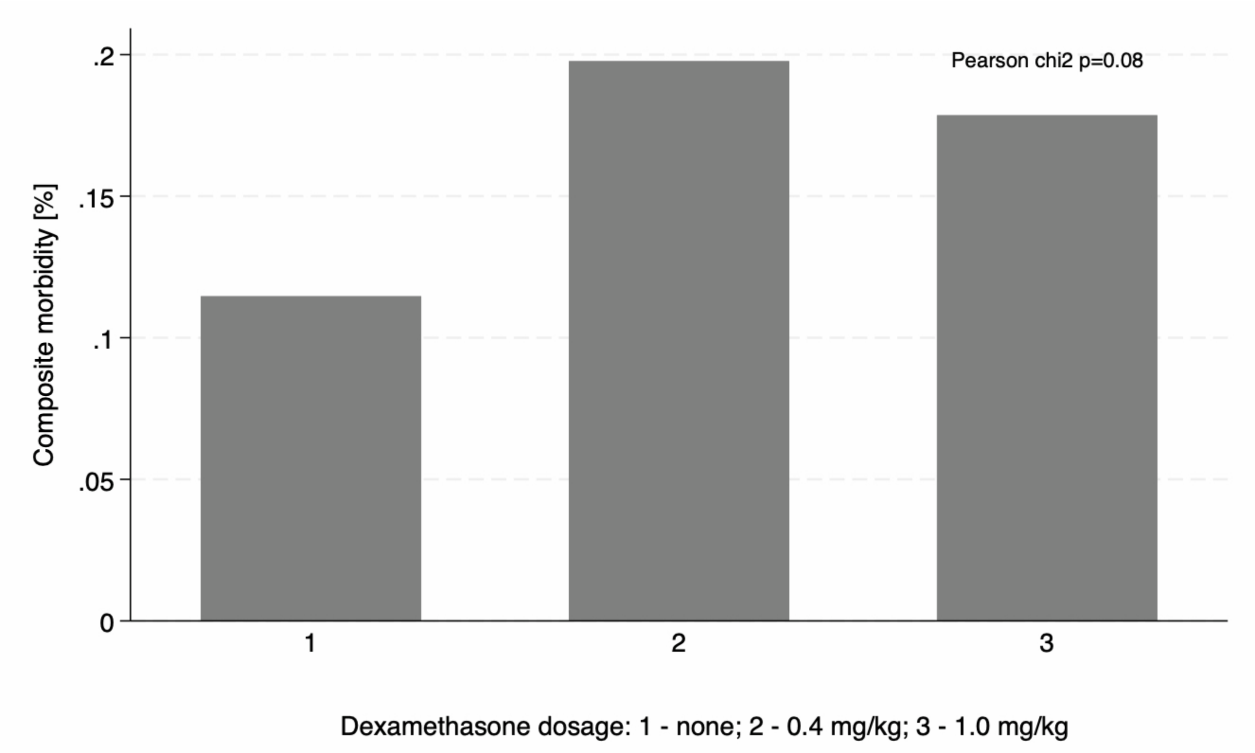

Figure S4

CRP trajectory correlation heatmap

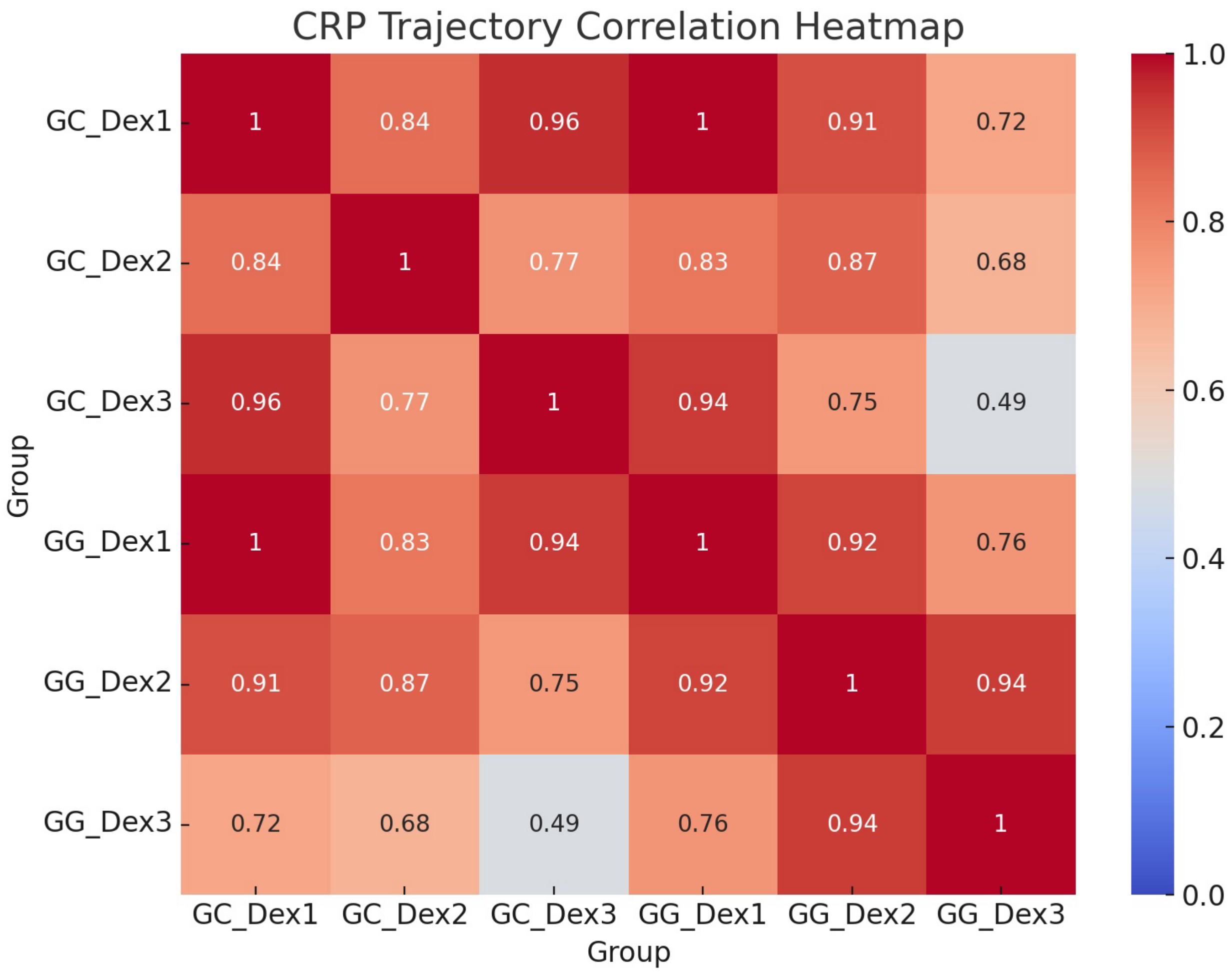

Statistical analysis plan

Data quality control:

1) Check of data format
2) Check for outliers

Outcome analysis:

3) Analysis of distribution of continuous data
4) Analysis of association between CRP1 and *CRP* rs1800947 genotypes (2 groups) – crude
5) Analysis of association between CRP1 and dexamethasone doses (3 groups)
6) Stratified analysis of association between CRP1 and *CRP* rs1800947 in dexamethasone dose strata.
7) Multivariate linear regression model of variables associated with CRP1.

Longitudinal analysis:

8) Selection of patients with at least 3 CRP measurements within first 4 postoperative days.
9) Analysis of CRP trajectories between *CRP* rs1800947 genotypes stratified for dexamethasone doses.
10) Similarity heat-map of trajectories.

Secondary outcomes analysis:

11) Associations with CRP1, *CRP* rs1800947 genotypes and dexamethasone use.
12) Analysis of association between four trajectory clusters and secondary outcomes.

The study sample is too small for sensitivity analysis or adjustment for further confounding variables.

Table S6 (Gallo et al. [2011] PLoS Med; doi:10.1371/journal.pmed.1001117)

Association between CRP rs1800947 genotypes, dexamethasone, postoperative CRP and morbidity in adult cardiac surgical patients – a hypothesis-generating analysis of the INFLACOR trial.

The Strengthening the Reporting Observational studies in Epidemiology – Molecular Epidemiology (STROBE-ME) Reporting Recommendations: Extended from STROBE statement				
**Item**	**Item number**	**STROBE Guidelines**	**Extension for Molecular Epidemiology Studies (STROBE-ME)**	
**Title and abstract**	1	(a) Indicate the study's design with a commonly used term in the title or the abstract	**ME-1** State the use of specific biomarker(s) in the title and/or in the abstract if they contribute substantially to the findings	Yes, p 1
		(b) Provide in the abstract an informative and balanced summary of what was done and what was found		Yes, p 3
**Introduction**				
Background rationale	2	Explain the scientific background and rationale for the investigation being reported	**ME-2** Explain in the scientific background of the study how/why the specific biomarker(s) have been chosen, potentially among many others (e.g., others are studied but reported elsewhere, or not studied at all)	Yes, p 6-9
Objectives	3	State specific objectives, including any pre-specified hypotheses	**ME-3** *A priori* hypothesis: if one or more biomarkers are used as proxy measures, state the *a priori* hypothesis on the expected values of the biomarker(s)	Yes, p 9
**Methods**				
Study design	4	Present key elements of study design early in the paper	**ME-4** Describe the special study designs for molecular epidemiology (in particular nested case/control and case/cohort) and how they were implemented	Yes, p 8-11
Biological sample collection			**ME-4.1** Report on the setting of the biological sample collection; amount of sample; nature of collecting procedures; participant conditions; time between sample collection and relevant clinical or physiological endpoints.	Yes, p 10-11
Biological sample storage			**ME-4.2** Describe sample processing (centrifugation, timing, additives, etc).	Yes, Ref 39
Biological sample processing			**ME-4.3** Describe sample storage until biomarker analysis (storage, thawing, manipulation, etc).	Yes, Ref 39
Biomarker biochemical characteristics			**ME-4.4** Report the half-life of the biomarker, and chemical and physical characteristics (e.g., solubility).	n/a
Setting	5	Describe the setting, locations, and relevant dates, including periods of recruitment, exposure, follow-up, and data collection		Yes, p 10
Participants	6	(a) Cohort study—Give the eligibility criteria, and the sources and methods of selection of participants. Describe methods of follow-up<br>Case-control study—Give the eligibility criteria, and the sources and methods of case ascertainment and control selection. Give the rationale for the choice of cases and controls<br>Cross-sectional study—Give the eligibility criteria, and the sources and methods of selection of participants	**ME-6** Report any habit, clinical conditions, physiological factor, or working or living condition that might affect the characteristics or concentrations of the biomarker	Yes / n/a p 9
		(b) Cohort study—For matched studies, give matching criteria and number of exposed and unexposed<br>Case-control study—For matched studies, give matching criteria and the number of controls per case		p. 13
Variables	7	Clearly define all outcomes, exposures, predictors, potential confounders, and effect modifiers. Give diagnostic criteria, if applicable		Yes p 10-11, Tab S3
Data source/measurement	8	For each variable of interest, give sources of data and details of methods of assessment (measurement).<br>Describe comparability of assessment methods if there is more than one group	**ME-8** Laboratory methods: report type of assay used, detection limit, quantity of biological sample used, outliers, timing in the assay procedures (when applicable) and calibration procedures or any standard used	n/a
Bias	9	Describe any efforts to address potential sources of bias		Yes p 11-12
Study size	10	Explain how the study size was arrived at		Yes p 12

Quantitative variables	11	Explain how quantitative variables were handled in the analyses. If applicable, describe which groupings were chosen, and why		Yes, p 12-13
Statistical methods	12	(a) Describe all statistical methods, including those used to control for confounding	**ME-12** Describe how biomarkers were introduced into statistical models	Yes, p 12-13
		(b) Describe any methods used to examine subgroups and interactions		Yes, p 10-11
		(c) Explain how missing data were addressed		Yes, p 12-13
		(d) Cohort study—If applicable, explain how loss to follow-up was addressed<br>Case-control study—If applicable, explain how matching of cases and controls was addressed<br>Cross-sectional study—If applicable, describe analytical methods taking account of sampling strategy		Yes, p 18-19
		(e) Describe any sensitivity analyses		n/a
Validity/reliability of measurement and internal/external validation			**ME-12.1** Report on the validity and reliability of measurement of the biomarker(s) coming from the literature and any internal or external validation used in the study.	n/a
**Results**				
Participants	13	(a) Report the numbers of individuals at each stage of the study—e.g., numbers potentially eligible, examined for eligibility, con-<br>firmed eligible, included in the study, completing follow-up, and analysed	**ME-13** Give reason for loss of biological samples at each stage	Yes, p 14
		(b) Give reasons for non-participation at each stage		Yes, p 14
		(c) Consider use of a flow diagram		Yes, p 14
Descriptive data	14	(a) Give characteristics of study participants (e.g., demographic, clinical, social) and information on exposures and potential con-founders		Yes Table 1, S1, S2
		(b) Indicate the number of participants with missing data for each variable of interest		n/a
		(c) Cohort study—Summarise follow-up time (e.g., average and total amount)		Yes, p 20
Distribution of biomarker measurement			**ME-14.1** Give the distribution of the biomarker measurement (including mean, median, range, and variance)	Yes, p 16
Outcome data	15	Cohort study—Report numbers of outcome events or summary measures over time<br>Case-control study—Report numbers in each exposure category, or summary measures of exposure<br>Cross-sectional study—Report numbers of outcome events or summary measures		Yes, p 20-22
Main results	16	(a) Give unadjusted estimates and, if applicable, confounder-adjusted estimates and their precision (e.g., 95% confidence interval).<br>Make clear which confounders were adjusted for and why they were included		Yes, p 16, Table 4
		(b) Report category boundaries when continuous variables were categorized		Yes, p 18-19
		(c) If relevant, consider translating estimates of relative risk into absolute risk for a meaningful time period		n/a
Other analyses	17	Report other analyses done—e.g., analyses of subgroups and interactions, and sensitivity analyses		Yes, p 20-22
**Discussion**				
Key results	18	Summarise key results with reference to study objectives		Yes, p 23
Limitations	19	Discuss limitations of the study, taking into account sources of potential bias or imprecision. Discuss both direction and magnitude<br>of any potential bias	**ME-19** Describe main limitations in laboratory procedures	n/a
Interpretation	20	Give a cautious overall interpretation of results considering objectives, limitations, multiplicity of analyses, results from similar studies, and other relevant evidence	**ME-20** Give an interpretation of results in terms of *a-priori* biological plausibility	p 24-26
Generalisability	21	Discuss the generalisability (external validity) of the study results		p 26-27
**Other information**				

Funding	22	Give the source of funding and the role of the funders for the present study and, if applicable, for the original study on which the present article is based		p 29
Ethics			**ME-22.1** Describe informed consent and approval from ethical committee(s). Specify whether samples were anonymous, anonymised or identifiable	p 10, 29

CONSORT 2025 checklist of information to include when reporting a randomised trial*

Section / Topic	No	CONSORT 2025 checklist item description	Reported on page no.
**Title and abstract**			
Title and structured abstract	1a	Identification as a ~~randomised trial~~ – **observational cohort trial**	p. 1
	1b	Structured summary of the trial design, methods, results, and conclusions	p. 3
**Open science**			
Trial registration	2	Name of trial registry, identifying number (with URL) and date of registration	p. 1
Protocol and statistical analysis plan	3	Where the trial protocol and statistical analysis plan can be accessed	p. 2, Supl. Mat.
Data sharing	4	Where and how the individual de-identified participant data (including data dictionary), statistical code and any other materials can be accessed	p. 29
Funding and conflicts of interest	5a	Sources of funding and other support (e.g., supply of drugs), and role of funders in the design, conduct, analysis and reporting of the trial	p. 29
	5b	Financial and other conflicts of interest of the manuscript authors	p. 29
**Introduction**			
Background and rationale	6	Scientific background and rationale	p. 6-9
Objectives	7	Specific objectives related to benefits and harms	p. 9
**Methods**			
Patient and public involvement	8	Details of patient or public involvement in the design, conduct and reporting of the trial	p. 10-11
Trial design	9	Description of trial design including type of trial (e.g., parallel group, crossover), allocation ratio, and framework (e.g., superiority, equivalence, non-inferiority, exploratory)	p. 10
Changes to trial protocol	10	Important changes to the trial after it commenced including any outcomes or analyses that were not prespecified, with reason	n/a
Trial setting	11	Settings (e.g., community, hospital) and locations (e.g., countries, sites) where the trial was conducted	p. 10
Eligibility criteria	12a	Eligibility criteria for participants	p. 10
	12b	If applicable, eligibility criteria for sites and for individuals delivering the interventions (e.g., surgeons, physiotherapists)	n/a

Intervention and comparator	13	Intervention and comparator with sufficient details to allow replication. If relevant, where additional materials describing the intervention and comparator (e.g., intervention manual) can be accessed	p. 10, 15
Outcomes	14	Pre-specified primary and secondary outcomes, including the specific measurement variable (e.g., systolic blood pressure), analysis metric (e.g., change from baseline, final value, time to event), method of aggregation (e.g., median, proportion), and time point for each outcome	p. 10-12
Harms	15	How harms were defined and assessed (e.g., systematically, non-systematically)	p. 11
Sample size	16a	How sample size was determined, including all assumptions supporting the sample size calculation	p. 12
	16b	Explanation of any interim analyses and stopping guidelines	n/a
Randomisation:			
Sequence generation	17a	Who generated the random allocation sequence and the method used	p. 8-9
	17b	Type of randomisation and details of any restriction (e.g., stratification, blocking and block size)	n/a
Allocation concealment mechanism	18	Mechanism used to implement the random allocation sequence (e.g., central computer/telephone; sequentially numbered, opaque, sealed containers), describing any steps to conceal the sequence until interventions were assigned	n/a
Implementation	19	Whether the personnel who enrolled and those who assigned participants to the interventions had access to the random allocation sequence	p. 8-9
Blinding	20a	Who was blinded after assignment to interventions (e.g., participants, care providers, outcome assessors, data analysts)	p. 11
	20b	If blinded, how blinding was achieved and description of the similarity of interventions	Suppl. Mat.
Statistical methods	21a	Statistical methods used to compare groups for primary and secondary outcomes, including harms	p. 11-12
	21b	Definition of who is included in each analysis (e.g., all randomised participants), and in which group	p. 10-12
	21c	How missing data were handled in the analysis	p. 12
	21d	Methods for any additional analyses (e.g., subgroup and sensitivity analyses), distinguishing prespecified from post-hoc	p. 12
**Results**			
Participant flow, including flow diagram	22a	For each group, the numbers of participants who were randomly assigned, received intended intervention, and were analysed for the primary outcome	p. 14
	22b	For each group, losses and exclusions after randomisation, together with reasons	p. 14
Recruitment	23a	Dates defining the periods of recruitment and follow-up for outcomes of benefits and harms	p. 10; n/a
	23b	If relevant, why the trial ended or was stopped	n/a

Intervention and comparator delivery	24a	Intervention and comparator as they were actually administered (e.g., where appropriate, who delivered the intervention/comparator, how participants adhered, whether they were delivered as intended [fidelity])	n/a
	24b	Concomitant care received during the trial for each group	n/a
Baseline data	25	A table showing baseline demographic and clinical characteristics for each group	p. 15, Suppl. Mat. P. 1-2
Numbers analysed, outcomes and estimation	26	For each primary and secondary outcome, by group:<br>● the number of participants included in the analysis<br>● the number of participants with available data at the outcome time point<br>● result for each group, and the estimated effect size and its precision (such as 95% confidence interval)<br>● for binary outcomes, presentation of both absolute and relative effect size	p. 16-22
Harms	27	All harms or unintended events in each group	p.21-22
Ancillary analyses	28	Any other analyses performed, including subgroup and sensitivity analyses, distinguishing pre-specified from post-hoc	n/a
**Discussion**			
Interpretation	29	Interpretation consistent with results, balancing benefits and harms, and considering other relevant evidence	p. 23-26
Limitations	30	Trial limitations, addressing sources of potential bias, imprecision, generalisability, and, if relevant, multiplicity of analyses	p. 27

*We strongly recommend reading this statement in conjunction with the CONSORT 2025 Explanation and Elaboration and/or the CONSORT 2025 Expanded Checklist for important clarifications on all the items. We also recommend reading relevant CONSORT extensions. See www.consort-spirit.org.

Citation: Hopewell S, Chan AW, Collins GS, Hróbjartsson A, Moher D, Schulz KF, et al. CONSORT 2025 Statement: updated guideline for reporting randomised trials. BMJ. 2025; 388:e081123. https://dx.doi.org/10.1136/bmj-2024-081123.

© 2025 Hopewell et al. This is an Open Access article distributed under the terms of the Creative Commons Attribution License (https://creativecommons.org/licenses/by/4.0/), which permits unrestricted use, distribution, and reproduction in any medium, provided the original work is properly cited.
