# Supplementary material for: Association between CRP rs1800947 genotypes, dexamethasone use, postoperative CRP level and morbidity in adult cardiac surgical patients in post-hoc analysis of the observational INFLACOR cohort trial
Source: Sci Rep. 2026 May 24;16:23754. doi: 10.1038/s41598-026-54801-9 (PMC13429688; doi:10.1038/s41598-026-54801-9)
Supplement: Supplementary file 2 — Supplementary Material 2 [file 41598_2026_54801_MOESM2_ESM.pdf]

***"INFLACOR — Clinical and Genetic Predictors of Inflammation-Related Complications After Heart Surgery"* - Research Protocol**

Part 1

1. Project Summary

Protocol title:
INFLACOR — Clinical and Genetic Predictors of Inflammation-Related Complications After Heart Surgery

Official title:
INFLACOR — *(INFLA-mmation, A-fter, C-ardiac, O-pe-R-ation)* — Evaluation of Selected Genetic Polymorphisms, Inflammatory Mediators, and Physiologic Parameters in the Prognosis of Postoperative Complications in Patients Undergoing Cardiopulmonary Bypass Cardiac Surgery.

Protocol identifying number:

ClinicalTrials.gov Identifier: **NCT01020409**

Study ID numbers: **N N403 1815 34; MUG grant G-35**

Project summary:

INFLACOR was a prospective observational cohort study designed to evaluate a clinically and economically effective diagnostic algorithm for predicting inflammatory response-related complications in adult patients undergoing first-time scheduled heart surgery with cardiopulmonary bypass. The study compared previously identified predictors of mortality and morbidity used in established cardiac surgical risk models, including EuroSCORE, CABDEAL, and Cleveland models, with new candidate predictors, including anamnestic, biochemical, genetic, and early postoperative clinical variables.

The study population consisted of adult patients aged 18 years or older who provided written informed consent and were scheduled for elective cardiac or ascending aorta surgery with cardiopulmonary bypass, with or without aortic cross-clamping. The planned and completed enrolment was 525 participants at the Medical University of Gdańsk, Academic Clinical Centre, Department of Cardiac Anaesthesiology, Gdańsk, Poland.

The primary outcome was the occurrence of clinical phenotypes of inflammatory response-related complications from postoperative day 2 until hospital discharge, including systemic inflammatory response syndrome, acute lung injury or acute respiratory distress syndrome, acute kidney injury, atrial fibrillation, postoperative psychosis, perioperative myocardial infarction or myocardial injury, and sepsis. The secondary outcome was all-cause in-hospital mortality from postoperative day 2 after surgery. Biospecimens including plasma, serum, and blood morphologic elements after centrifugation were retained frozen at −75°C, with DNA-containing samples stored for genetic analyses.

2. General Information

2.1 Protocol title

INFLACOR — Clinical and Genetic Predictors of Inflammation-Related Complications After Heart Surgery

2.2 Official title

INFLACOR — *(INFLA-mmation, A-fter, C-ardiac, O-pe-R-ation)* — Evaluation of Selected Genetic Polymorphisms, Inflammatory Mediators, and Physiologic Parameters in the Prognosis of Postoperative Complications in Patients Undergoing Cardiopulmonary Bypass Cardiac Surgery.

2.3 Protocol identifying number

ClinicalTrials.gov Identifier: NCT01020409

Study ID numbers: N N403 1815 34; MUG grant G-35

Acronym: INFLACOR

2.4 Protocol date and registry dates

Study start date: October 2009

Primary completion date: April 2011

Study completion date: November 2012

First posted on ClinicalTrials.gov: 25 November 2009

PRS receipt release date: 15 May 2015

Last update posted: 15 November 2016

2.5 Sponsor and funder

Sponsor:

Medical University of Gdańsk, Poland

Collaborator / funding authority:

Ministry of Science and Higher Education, Poland

Funder type:

Other

2.6 Responsible party

Responsible Party:

Maciej M. Kowalik, Dr., Medical University of Gdańsk

2.7 Investigators

Study Chair:

Romuald Lango, MD, PhD

Medical University of Gdańsk

Study Director:

Maciej M. Kowalik, MD, PhD

Medical University of Gdańsk

2.8 Research site

Medical University of Gdańsk

Academic Clinical Centre

Department of Cardiac Anaesthesiology

Gdańsk, PL-80-211

Poland

2.9 Clinical laboratories, technical departments, and institutions involved

The study involved biospecimen collection and retention, including plasma, serum, and blood morphologic elements after centrifugation, frozen at −75°C, with samples containing DNA. Samples were obtained, centrifugated and deep frozen at the Department of Cardiac Anaesthesiology. Biochemical analyses were performed at the Central Laboratory of the University Clinical Centre Gdańsk. Serum samples for cytokines analyses were processed at the Flow Cytometry Laboratory of The Department of Clinical Chemistry and Biochemistry, Medical University of Gdańsk. Genetic sequencing and analyses were performed at The Department of Biology and Genetics, Medical University of Gdańsk.

3. Rationale and Background Information

Cardiac surgery with cardiopulmonary bypass is associated with activation of systemic inflammatory pathways. This inflammatory response may contribute to clinically relevant postoperative complications, including systemic inflammatory response syndrome, acute lung injury or acute respiratory distress syndrome, acute kidney injury, atrial fibrillation, postoperative psychosis, perioperative myocardial infarction or myocardial injury, sepsis, and death during hospitalisation. Several risk prediction systems, including EuroSCORE, CABDEAL, and Cleveland models, have been used to estimate morbidity and mortality after cardiac surgery. However, these models are primarily based on clinical risk factors and may not fully account for inflammatory, biochemical, genetic, or early postoperative physiological predictors. INFLACOR was designed to compare established risk predictors with new candidate variables, including anamnestic factors, inflammatory biomarkers, genetic polymorphisms, and early postoperative clinical indicators.

The study addresses the clinical need for improved prediction of inflammation-related complications after cardiac surgery with cardiopulmonary bypass. A clinically and economically effective diagnostic algorithm may help identify patients at increased risk and support perioperative risk stratification, postoperative surveillance, and potentially targeted preventive strategies.

The study specifically evaluates selected genetic polymorphisms, inflammatory mediators, and physiological parameters in relation to postoperative inflammatory response-related complications.

4. References

1. Butler J, Baigrie RJ, Parker D, Chong JL, Shale DJ, Pillai R, Westaby S, Rocker GM. Systemic inflammatory responses to cardiopulmonary bypass: a pilot study of the effects of pentoxifylline. *Respir Med.* 1993;87(4):285–288.

2. Asimakopoulos G. Mechanisms of the systemic inflammatory response. *Perfusion.* 1999;14(4):269–277.

3. Higgins TL, Estafanous FG, Loop FD, Beck GJ, Blum JM, Paranandi L. Stratification of morbidity and mortality outcome by preoperative risk factors in coronary artery bypass patients. A clinical severity score. *JAMA.*1992;267(17):2344–2348.

4. Nashef SA, Roques F, Michel P, Gauducheau E, Lemeshow S, Salamon R. European system for cardiac operative risk evaluation — EuroSCORE. *Eur J Cardiothorac Surg.* 1999;16(1):9–13.

5. Kurki TS, Järvinen O, Kataja MJ, Laurikka J, Tarkka M. Performance of three preoperative risk indices: CABDEAL, EuroSCORE and Cleveland models in a prospective coronary bypass database. *Eur J Cardiothorac Surg.* 2002;21(3):406–410.

6. Butler J, Chong GL, Baigrie RJ, Pillai R, Westaby S, Rocker GM. Cytokine responses to cardiopulmonary bypass with membrane and bubble oxygenation. *Ann Thorac Surg.* 1992;53(5):833–838.

7. Lamm G, Auer J, Weber T, Berent R, Ng C, Eber B. Postoperative white blood cell count predicts atrial fibrillation after cardiac surgery. *J Cardiothorac Vasc Anesth.* 2006;20(1):51–56.

8. Podgoreanu MV, White WD, Morris RW, Mathew JP, Stafford-Smith M, Welsby IJ, Grocott HP, Milano CA, Newman MF, Schwinn DA; PEGASUS Investigative Team. Inflammatory gene polymorphisms and risk of postoperative myocardial infarction after cardiac surgery. *Circulation.* 2006;114(1 Suppl):I275–I281.

9. Gaudino M, Di Castelnuovo A, Zamparelli R, Andreotti F, Burzotta F, Iacoviello L, Glieca F, Alessandrini F, Nasso G, Donati MB, Maseri A, Schiavello R, Possati G. Genetic control of postoperative systemic inflammatory reaction and pulmonary and renal complications after coronary artery surgery. *J Thorac Cardiovasc Surg.*2003;126(4):1107–1112.

10. Bone RC, Balk RA, Cerra FB, Dellinger RP, Fein AM, Knaus WA, Schein RM, Sibbald WJ. Definitions for sepsis and organ failure and guidelines for the use of innovative therapies in sepsis. *Chest.* 1992;101(6):1644–1655.

11. Levy MM, Fink MP, Marshall JC, Abraham E, Angus D, Cook D, Cohen J, Opal SM, Vincent JL, Ramsay G; SCCM/ESICM/ACCP/ATS/SIS. 2001 International Sepsis Definitions Conference. *Crit Care Med.*2003;31(4):1250–1256.

12. Mitchell JD, Grocott HP, Phillips-Bute B, Mathew JP, Newman MF, Bar-Yosef S. Cytokine secretion after cardiac surgery and its relationship to postoperative fever. *Cytokine.* 2007;38(1):37–42.

13. Bellomo R, Kellum JA, Ronco C. Defining and classifying acute renal failure: from advocacy to consensus and validation of the RIFLE criteria. *Intensive Care Med.* 2007;33(3):409–413.

14. Bernard GR, Artigas A, Brigham KL, Carlet J, Falke K, Hudson L, Lamy M, Legall JR, Morris A, Spragg R. The American-European Consensus Conference on ARDS: definitions, mechanisms, relevant outcomes, and clinical trial coordination. *Am J Respir Crit Care Med.* 1994;149(3 Pt 1):818–824.

15. Knaus WA, Wagner DP, Draper EA, Zimmerman JE, Bergner M, Bastos PG, Sirio CA, Murphy DJ, Lotring T, Damiano A, et al. The APACHE III prognostic system. *Chest.* 1991;100(6):1619–1636.
16. van Gemert LA, Schuurmans MJ. The Neecham Confusion Scale and the Delirium Observation Screening Scale: capacity to discriminate and ease of use in clinical practice. *BMC Nurs.* 2007;6:3.
17. Thygesen K, Alpert JS, White HD; Joint ESC/ACCF/AHA/WHF Task Force. Universal definition of myocardial infarction. *Eur Heart J.* 2007;28(20):2525–2538.

5. Study Goals and Objectives

5.1 General goal

The general goal of the study was to evaluate a clinically and economically effective diagnostic algorithm for the prediction of inflammatory response-related complications in patients undergoing heart surgery with the use of cardiopulmonary bypass.

5.2 Primary objective

To evaluate the predictive capability of selected established and novel candidate variables for clinical phenotypes of inflammatory response-related complications occurring after cardiac surgery, beginning from postoperative day 2 until hospital discharge.

The primary clinical phenotypes included: systemic inflammatory response syndrome (SIRS; acute lung injury (ALI) / acute respiratory distress syndrome (ARDS); acute kidney injury (AKI); atrial fibrillation; postoperative psychosis (delirium); perioperative myocardial infarction / myocardial injury; sepsis.

5.3 Secondary objective

To evaluate all-cause in-hospital mortality from postoperative day 2 after surgery.

5.4 Candidate predictor domains

The study compared predictors previously identified in risk prediction models, including EuroSCORE, CABDEAL, and Cleveland models, with new candidate variables grouped as follows:

5.4.1 Anamnestic variables

- recent tooth extractions (up to 60 days);
- chronic inflammatory diseases;
- specific drug use.

5.4.2 Biochemical variables

- C-reactive protein;
- interleukin-6;
- tumour necrosis factor alpha;
- routine biochemical blood tests (eGFR, blood morphology, prothrombin time, INR, creatinine, blood urea nitrogen, phosphokinase MB fraction, high-sensitive troponin I)

5.4.3 Genetic variables

- single nucleotide polymorphisms of 10 genes associated with inflammatory response:

IL6 rs1800796, *LBP1* rs2232582, *ICAM1* rs5498, *CRP* rs1800947, *NOD2* rs2066844, *TNF* rs1800629, *MASP2* rs2273346, *SELE* rs1805193, *NOS3* rs1799983, *TLR4* rs4986790.

5.4.4 Early postoperative clinical variables

- systemic inflammatory response syndrome on postoperative day 1;
- APACHE III score on postoperative day 1.
- Clinical criteria of AKI, pneumonia, sepsis, myocardial infarction, stroke, new-onset atrial fibrillation, delirium.

6. Study Design

6.1 Type of study

Prospective observational cohort study.

6.2 Study classification

Study type: Observational

Observational model: Cohort

Time perspective: Prospective

Sampling method: Probability sample

6.3 Study setting

Single-centre study conducted at Medical University of Gdańsk, Academic Clinical Centre, Department of Cardiac Anaesthesiology, Gdańsk, PL-80-211, Poland

6.4 Study population

Adult patients undergoing first-time scheduled heart surgery with the use of cardiopulmonary bypass.

6.5 Group / cohort

CPB cardiac surgery cohort

Adult patients who signed informed consent and underwent first-time scheduled heart surgery with cardiopulmonary bypass.

6.6 Procedure / exposure

Procedure/Surgery:

Cardiac surgery with cardiopulmonary bypass use.

Description:

Heart or ascending aorta surgery performed with the use of cardiopulmonary bypass, with or without aortic cross-clamping.

Other names:

- heart valve surgery;
- open heart surgery.

6.7 Eligibility criteria

Inclusion criteria

Participants were eligible if they met all of the following criteria:

1. Adult patients aged 18 years or older.
2. Given and signed informed consent.
3. No previous cardiac surgery with opening of the pericardium.
4. Scheduled for elective cardiac surgery with the use of cardiopulmonary bypass.

Exclusion criteria

Participants were excluded if they met any of the following criteria:

1. Previous cardiac surgery with opening of the pericardium.
2. Consent refused or not given.

6.8 Sex and age

Ages eligible for study: 18 years and older

Sexes eligible for study: Both

6.9 Healthy volunteers

The ClinicalTrials.gov record states: **Accepts healthy volunteers: No.**

6.10 Enrolment

Enrolment: 525 participants

6.11 Study duration

Study start date: October 2009

Primary completion date: April 2011

Study completion date: November 2012

Approximate duration from study start to primary completion: 18 months.

Approximate total duration from study start to completion: 37 months.

6.12 Withdrawal criteria

Withdrawal criteria:

1. withdrawal of consent,
2. inability to complete required data collection,
3. other circumstances in which participant data or biospecimens would no longer be used.

7. Methodology

7.1 Overview

INFLACOR was a prospective observational cohort study of adult patients undergoing first-time scheduled heart surgery with cardiopulmonary bypass. The study evaluates whether selected clinical, anamnestic, biochemical, genetic, and postoperative physiological variables can predict inflammatory response-related complications after cardiac surgery.

7.2 Clinical procedure

All participants underwent heart or ascending aorta surgery with the use of cardiopulmonary bypass, with or without aortic cross-clamping, as part of standard clinical care. Because the study was observational, cardiac surgery with cardiopulmonary bypass represents the clinical exposure or operative context rather than an experimental intervention.

7.3 Baseline and candidate predictor assessment

The study evaluated predictors from four main domains:

7.3.1 Anamnestic predictors

- recent tooth extractions (<60 days);
- chronic inflammatory diseases (ie: SLE, rheumatoid arthritis and other; formal criteria: therapy with immunosuppressive drugs including corticosteroids, azathioprine and other);
- specific drug use (betablockers, ACE/ARB, aldosterone inhibitors, diuretics, statins, oral hypoglycemics)

7.3.2 Biochemical predictors on Postoperative Day 1

- C-reactive protein;
- interleukin-6;
- E-selctin;
- ICAM1
- tumour necrosis factor alpha.

The levels of interleukin 6 (IL-6), intercellular adhesion molecule 1 (ICAM1), soluble endothelial E-selectin (E-SEL), and tumor necrosis factor α (TNFα) in the deep-frozen serum samples that were obtained three hours after admission to the postoperative ICU were measured using flow cytometry bead-based assays using a Becton Dickinson FACSCanto II flow cytometer (BD Biosciences, San Diego, CA, USA) in conjunction with the following assays (CBA; Becton Dickinson Biosciences, San Diego, CA, USA): HU IL-6 CBA Flex Set Bead A7; HU TNF CBA Flex Set C4; HU SCD54 (ICAM1) CBA Flex Set 100TST A4; and HU SOLBL CD62E CBA Flex Set 100 T D9. The samples were acquired and prepared according to the manufacturer's instructions.

7.3.3 Genetic predictors

Single nucleotide polymorphisms (SNPs) of 10 genes associated with inflammatory response: *IL6* rs1800796, *LBP1* rs2232582, *ICAM1* rs5498, *CRP* rs1800947, *NOD2* rs2066844, *TNF* rs1800629, *MASP2* rs2273346, *SELE* rs1805193, *NOS3* rs1799983, *TLR4* rs4986790.

The blood samples for genotype analyses were obtained 3 hours after admission to the postoperative unit. The blood cells were separated from the plasma/serum via cooled centrifugation (5 min, 3,000 rpm, 4°C). The serum or plasma was aliquoted manually and stored at –80°C. Genomic DNA was extracted from the leukocytes using a Genomic Midi AX kit (A&A Biotechnology, Poland) according to the manufacturer's protocol. The SNVs were amplified in two multiplex PCRs. The genotypes were analyzed using a single base primer extension assay with a SNaPshot® Multiplex Kit (Life Technologies, Foster City, CA, USA) according to the manufacturer's protocol. All the primers

(sequences are available upon request) were designed using Primer 3 (http://frodo.wi.mit.edu/). Capillary electrophoresis was performed on an Applied Biosystems 3130 Genetic Analyzer, and the obtained electrophoregrams were analyzed using GeneMapper version 4.0 (Life Technologies, Foster City, CA, USA). The SNV genotypes of the selected samples were confirmed with an independent PCR followed by bidirectional sequencing using an Applied Biosystems 3130 Genetic Analyzer and the BigDye Terminator v3.1 Cycle Sequencing Kit (Life Technologies, Foster City, CA, USA). The sequences were analyzed using Sequencher v.4.10.1 DNA (Gene Codes Corporation, Ann Arbor, MI, USA). Data were checked for Hardy-Weinberg equilibrium.

7.3.4 Early postoperative clinical predictors

- systemic inflammatory response syndrome on postoperative day 1;
- APACHE III score on postoperative day 1.

7.4 Biospecimen collection and retention

The study included retention of samples with DNA. The retained biological material included samples obtained 3 and 18 hours after patients admission to ICU: plasma 2 x 2 mL; serum 2 x 1,5 mL; blood morphologic elements after centrifugation 2 x 1,5 mL. Samples were frozen to **−80°C**. Samples were cooled at 4°C and centrifugated for 5 min, at 3,000 rpm.

7.5 Outcome assessment

7.5.1 Primary outcome

The primary outcome consisted of clinical phenotypes of inflammatory response-related complications occurring between postoperative day 2 and hospital discharge. These included:

1. systemic inflammatory response syndrome;
2. acute lung injury / acute respiratory distress syndrome;
3. acute kidney injury;
4. atrial fibrillation;
5. postoperative psychosis;
6. perioperative myocardial infarction / myocardial injury;
7. sepsis.

7.5.2 Secondary outcome

The secondary outcome was:

- all-cause in-hospital mortality from postoperative day 2 after operation at 30 days, 1 year and 5 years.

7.6 Diagnostic definitions

The ClinicalTrials.gov PRS receipt includes references supporting definitions of sepsis, acute kidney injury, ARDS, APACHE III, postoperative confusion/delirium screening, and myocardial infarction. The operational diagnostic criteria were:

- SIRS criteria: Min 2/4 criteria: 1. Sinus tachycardia > 90'; 2. Hyperventilation (spontaneous breathing) – Rr > 20' or $PaCO_2$ < 32 mmHg; 3. Temperature > 38.0 or < 36.0 C; 4. WBC < 4.0 G/L or 12.0 G/L or immature granular forms > 10%.
- ALI / ARDS criteria: ALI - acute hypoxemia with PaO_2:FiO_2 ≤300 mmHg + bilateral edematous/inflammatory changes (also very subtle) on chest X-ray consistent with pulmonary edema + no clinical evidence of elevated LA pressure or PAWP ≤18 mmHg; ARDS: as above, but with PaO_2:FiO_2 ≤200 mmHg.
- AKI criteria (RIFLE-criteria): R - creatinine x 1.5, or a decrease in GFR > 25%; or diuresis < 0.5 ml/kg/h x 6 hrs; I - creatinine x 2, or a decrease in GFR > 50%; or diuresis < 0.5 ml/kg/h x 12 hrs; F - creatinine x 3, or a decrease in GFR > 75%; or diuresis < 0.3 ml/kg/h x 24 hrs, or anuria x 12 hrs.
- New-onset atrial fibrillation definition: only episodes which required treatment, duration of episode recorded.
- postoperative delirium (psychosis): Delirium Observatio Screening Scale (DOSS) >4 requiring therapy with sedatives or neuroleptics.
- myocardial infarction or myocardial injury definition: 1. Clinical symptoms – angina, nausea, syncope, or 2. ECG in the absence of LVH and LBBB: (1) ST elevation – new ST elevation in the J pt in at least two adjacent leads ≥0.2 mV (men) / ≥0.15 mV (women) in V2-V3 and/or ≥0.1 mV in other leads, (2) ST depression and T wave changes – new horizontal or descending ST depression ≥0.05 mV in two adjacent leads; and/or T wave inversion ≥0.1 mV in two adjacent leads with a dominant R wave or R/S ratio >1, (3) new LBBB; or 3. Echocardiography – new contractility abnormalities; **and** 4. CPK-MB: women > 17, men > 36 ng/ml, troponin I: women > 0.045, men > 0.11 ng/ml.
- sepsis definition and grading: 1. SIRS – as above; 2. severe sepsis – SIRS + hypotension (SAP < 90 mmHg), organ failure or hypoperfusion (i.e., lactic acidosis, prolonged capillary refill time > 2 seconds, oliguria, acute disturbances of consciousness, arterial hypoxemia – PaO_2/FiO_2 <300, creatinine increase of 0.5 mg/dL, non-drug-induced coagulation disorders – INR > 1.5, obstruction – lack of peristalsis, thrombocytopenia – PLT < 100 K/μL, hyperbilirubinemia – total bilirubin > 4 mg/dL); 3. septic shock – hypotension refractory to fluid resuscitation (requiring the initiation/increase of amines).

7.7 Randomisation and blinding

Randomisation was not applicable because the study was observational. Outcome assessors were not blinded. Laboratory personnel and genetic analysts were blinded to clinical data and outcomes.

7.8 Graphic outline of study design and procedures

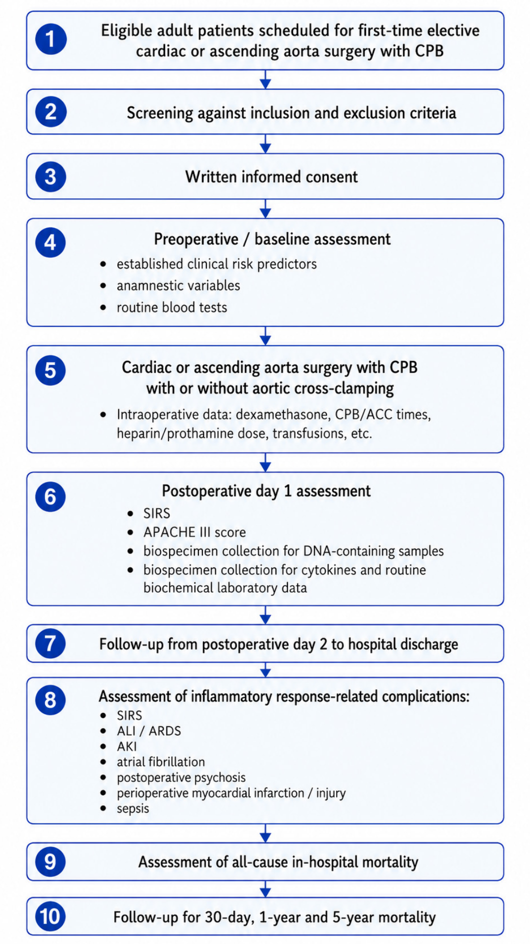

8. Safety Considerations

The study was observational and did not involve an experimental drug, device, or vaccine. The main clinical procedure — cardiac surgery with cardiopulmonary bypass — was performed as part of standard medical care. Potential research-specific safety and ethical considerations included: risks associated with blood sampling; confidentiality of clinical and genetic data; storage and future use of DNA-containing samples; appropriate handling of clinically relevant incidental findings, if any; minimisation of interference with standard perioperative care.

The primary and secondary outcome measures were designated as **not safety issues**.

Other safety issues, including adverse event recording procedures, serious adverse event reporting procedures, safety monitoring responsibilities were subject to routine in-hospital reporting and QMM policy. For this study purposes a Data Safety Monitoring Board, procedures for reporting unexpected study-related risks, genetic counselling procedures were not required.

9. Follow-up

Participants were followed from surgery through hospital discharge for the occurrence of inflammatory response-related complications.

The primary outcome was assessed between postoperative day 2 and hospital discharge.

The secondary outcome — all-cause in-hospital mortality — was assessed from postoperative day 2 after surgery. After hospital discharge the patient were followed-up only for mortality at 30-days, 1-year and 5-years. Patients with incomplete follow-up / missing CRF's were excluded from analyses.

10. Data Management and Statistical Analysis

10.1 Data management

The study collected clinical, anamnestic, biochemical, genetic, and postoperative outcome data. Anamnestic, routine biochemical blood tests, intraoperative data, clinical outcomes were collected manually on case report forms. The study's database was prepared in licensed MS Access software. Data were entered and cross-reviewed by two trained data managers. Data were pseudoanonimisated upon input into the database upon unique study ID's. Linkage between clinical data and biospecimens was guaranteed upon unique study ID's. Access control to study data was warranted by physical (controlled room access) and IT (database login/password protection; database storage only on physical media) barriers. Data backup was performed every 30 days on physical media. Identification and correction of erroneous data included visually check of all entered data and verification against original CRF/HIS data in case of identified errors. No automated data safety check was performed.

10.2 Statistical analysis — general approach

The aim of the statistical analysis was to compare the predictive capability of previously identified risk predictors with new candidate variables for postoperative inflammatory response-related complications. Established predictors from previous models, including EuroSCORE, CABDEAL, and Cleveland models, are to be compared with: anamnestic variables; biochemical inflammatory markers; genetic polymorphisms; postoperative day 1 clinical variables. The outcomes of interest are inflammatory response-related clinical phenotypes occurring from postoperative day 2 onward and all-cause in-hospital mortality.

10.3 Suggested statistical analysis framework for completion of protocol

1. **Descriptive, univariate and multivariate analyses**

Baseline demographic, clinical, operative, biochemical, and genetic characteristics and univariate and multivariate analyses were summarised for the total cohort and according to occurrence of postoperative complications in a previous publication: Kowalik, M. M. *et al.* Clinical, biochemical and genetic risk factors for 30-day and 5-year mortality in 518 adult patients subjected to cardiopulmonary bypass during cardiac surgery - the INFLACOR study. *Acta Biochim Pol* **65**, 241–250 (2018). https://doi.org/10.18388/abp.2017_2361

10.4 Sample size

The sample size of the INFLACOR trial (n=525), a prospective observational cohort study, was determined on the basis of the following: 1) the duration of grant funding (3 years), 2) financial limits that restricted laboratory analyses to no more than 500 patients, 3) the operating volume of the research centre, and 4) an assumed drop-out rate of 5%.

11. Quality Assurance

The study uses defined eligibility criteria, prospective cohort design, and biospecimen retention at −75°C. The quality assurance plan included investigator training, standard operating procedures, Good Clinical Practice compliance, where applicable, procedures for informed consent documentation, data quality monitoring, laboratory quality control, genetic assay quality control, biospecimen storage monitoring, freezer temperature monitoring, source data verification, audit procedures, outcome adjudication procedures and handling of protocol deviations.

12. Expected Outcomes of the Study

The expected outcome of the study was the evaluation of a clinically and economically effective diagnostic algorithm for predicting inflammatory response-related complications after cardiac surgery with cardiopulmonary bypass. The study may contribute to: improved identification of patients at increased risk of inflammatory postoperative complications; better understanding of the relationship between genetic polymorphisms, inflammatory mediators, physiological parameters, and postoperative outcomes; refinement of perioperative risk stratification; potential development of more individualised postoperative monitoring strategies; comparison of established clinical risk models with expanded models incorporating inflammatory and genetic predictors.

13. Dissemination of Results and Publication Policy

Available information:

The study results were disseminated through peer-reviewed scientific publications and conference presentations. Authorship follows internationally accepted authorship criteria. The sponsor, funder, investigators, and collaborating units were acknowledged according to their contribution and applicable institutional policies. Up to date the study's results were published in 1 reviewed original report (*ABP*, 2018) and 7 oral presentations at the European Association of Cardiothoracic Anaesthesiologists annual congresses.

14. Duration of the Project

14.1 Overall timeline

Project phase	Date / duration
Study start	October 2009
Primary completion	April 2011
Study completion	November 2012
First ClinicalTrials.gov posting	25 November 2009
PRS receipt release date	15 May 2015
Last registry update	15 November 2016

14.2 Approximate duration

Approximate duration from study start to primary completion: **18 months**.

Approximate duration from study start to final completion: **37 months**.

14.3 Detailed month-by-month timeline

Detailed timeline:

- protocol preparation: IX-XII 2007
- ethics approval: 28.12.2007
- recruitment: X 2009 – III 2011
- surgery and perioperative data collection: X 2009 – III 2011
- biochemical analyses: X 2009 – III 2011
- genetic analyses: II 2010 -VI 2011
- outcome assessment: X 2009 – IV 2011
- data cleaning: 2011 – 2014
- statistical analysis: 2014 – now
- manuscript preparation: 2024-2025
- dissemination: since 2014 (includes 7 oral presentations at EACTA annual meetings, 1 reviewed original report)

15. Problems Anticipated

1. Incomplete clinical data: Some postoperative variables or complications may be incompletely documented.
2. Missing biochemical or genetic data: Biospecimen collection, storage, or assay failure may lead to missing laboratory or genetic variables.
3. Low event rates for selected outcomes: Some complications may occur infrequently, limiting statistical power for individual outcome models.
4. Heterogeneity of inflammatory phenotypes: SIRS, AKI, ARDS, atrial fibrillation, sepsis, and myocardial injury may have different mechanisms and predictors.
5. Risk of model overfitting: The number of candidate predictors, especially genetic variants, may be large relative to the number of outcome events.
6. Single-centre design: Generalisability to other centres may be limited.
7. Definitions of outcomes: Clinical phenotypes must be defined consistently to avoid misclassification.
8. Economic evaluation: Although we aimed clinical and economic effectiveness a formal health-economic analysis wasn't done yet.

Possible mitigation strategies include predefined outcome definitions, standardised data collection, careful handling of missing data, conservative model-building strategies, and transparent reporting of limitations.

16. Project Management

16.1 Sponsor

Medical University of Gdańsk

16.2 Collaborator / funding authority

Ministry of Science and Higher Education, Poland

16.3 Study leadership

Study Chair:

Romuald Lango, MD, PhD

Medical University of Gdańsk

Study Director:

Maciej M. Kowalik, MD, PhD

Medical University of Gdańsk

Principal Investigator:

Jan Rogowski, MD, PhD

Medical University of Gdańsk

16.4 Responsible party

Maciej M. Kowalik, Dr., Medical University of Gdańsk

16.5 Research site

Medical University of Gdańsk

Academic Clinical Centre

Department of Cardiac Anaesthesiology

Gdańsk, PL-80-211, Poland

16.6 Roles and responsibilities

- participant screening: Maciej M. Kowalik, Romuald Lango, Maciej Brzeziński, Andrzej Łoś, Rafał Pawlaczyk, Dariusz Jagieak, Piotr Siondalski, Jan Rogowski
- informed consent: Maciej M. Kowalik, Romuald Lango, Violetta Musiał-Świątkiewicz, Barbara Treszer, Ewa Siondalska, Mariola Stefaniak, Paweł Mroziński.
- perioperative clinical data collection: Maciej M. Kowalik, Romuald Lango, Andrzej Łoś, Maciej Brzeziński, Piotr Siondalski, Rafał Pawlaczyk,
- biospecimen collection: Maciej M. Kowalik, nursing staff
- laboratory analysis: Krzysztof Lewandowski, Małgorzata Smyll
- genetic testing: Magdalena Chmara, Monika Żuk
- outcome adjudication: Maciej M. Kowalik, Romuald Lango, Rafał Pawlaczyk, Dariusz Jagielak, Maciej Brzeziński, Andrzej Łoś.
- data management: Maciej Kowalik, Aleksandra Biedrzycka, Magdalena Łasińska-Kowara
- statistical analysis in all publications: Maciej Kowalik, Romuald Lango
- manuscript preparation (published and under review): Maciej Kowalik, Romuald Lango, Jan Rogowski, Maciej Brzeziński, Piotr Siondalski, Krzysztof Lewandowski, Magdalena Chmara.
- archive and data protection: Maciej Kowalik, Romuald Lango, Maciej Brzeziński.

17. Ethics

17.1 Ethical nature of the study

The study was an observational cohort study involving adult cardiac surgical patients undergoing procedures as part of standard clinical care. The research includes collection and analysis of clinical data, biochemical inflammatory markers, and genetic polymorphisms, as well as storage of DNA-containing biospecimens.

17.2 Informed consent

The eligibility criteria required that participants gave and signed informed consent. Consent refused or not given is an exclusion criterion.

17.3 Ethical issues

Important ethical issues about which the participants were informed and towards which they gave their consent include: inclusion of surgical patients undergoing major cardiac procedures; collection and storage of biological samples containing DNA; analysis of genetic polymorphisms; confidentiality of clinical and genetic data; appropriate coding or pseudonymisation of samples and data; participant right to refuse participation; participant right to withdraw consent; governance of future use of stored samples.

17.4 Ethics approval

Name of the ethics committee: Niezależna Komisja Bioetyczna ds. Badań Naukowych przy Akademii Medycznej w Gdańsku (Independent Bioethical Comission for Scientific Research at the Medical University of Gdańsk)

Approval number: NKEBN/358/2007

Approval date: 28.12.2007

17.5 Confidentiality and data protection

Procedures for maintaining confidentiality and protecting personal and genetic data included physical barriers (CRFs stored in a limited access locked room and locked cabinet). Genetec data were pseudoanonimized. At no stage of genetic testing personal data were linked with genetic tests.

18. Informed Consent Forms

The informed consent forms, including separate consent for genetic testing with patient information sheet (in Polish) are available upon request to the study's director (M.M. Kowalik).

Part 2

19. Budget

The budget documents including costs of personnel, patient screening and recruitment, clinical data collection, biochemical assays, genetic analyses, biospecimen processing and storage, database development and maintenance, statistical analysis, administrative support, publication and dissemination were reported to the sponsoring authorities (Medical University of Gdańsk and Polish Ministry of Science and Higher Education) and are available on request.

20. Other Support for the Project

20.1 Available support

Sponsor:

Medical University of Gdańsk

Collaborator / funding authority:

Ministry of Science and Higher Education, Poland

Study ID numbers:

N N403 1815 34

MUG grant G-35

Health authority:

Poland: Ministry of Science and Higher Education

21. Collaboration with Other Scientists or Research Institutions

21.1 Available information

The study was sponsored by the Medical University of Gdańsk and the Ministry of Science and Higher Education, Poland.

The study was conducted at:

Medical University of Gdańsk

Academic Clinical Centre

Department of Cardiac Anaesthesiology

Gdańsk, Poland

22. Links to Other Projects

None

23. Curriculum Vitae of Investigators

CVs of Romuald Lango, MD, PhD, Maciej M. Kowalik, MD, PhD, Jan Rogowski, MD, PhD and other investigators are available on request.

24. Other Research Activities of the Investigators

Available upon request.

25. Financing and Insurance

25.1 Financing

The study was sponsored by the Medical University of Gdańsk and the Ministry of Science and Higher Education, Poland, under study ID numbers N N403 1815 34 and MUG grant G-35.

25.2 Insurance

This study was not covered by insurance.
